# The *N_e_*/*N* ratio in applied conservation

**DOI:** 10.1111/eva.13695

**Published:** 2024-05-08

**Authors:** Robin S. Waples

**Affiliations:** ^1^ School of Aquatic and Fishery Sciences University of Washington Seattle Washington USA

**Keywords:** age structure, effective population size, genetic drift, inbreeding, spatial structure

## Abstract

Recent developments within the IUCN and the Convention on Biological Diversity have affirmed the increasingly key role that effective population size (*N*_*e*_) and the effective size: census size ratio (*N*_*e*_/*N*) play in applied conservation and management of global biodiversity. This paper reviews and synthesizes information regarding the definition of *N*_*e*_ and demographic and genetic methods for estimating effective size, census size, and their ratio. Emphasis is on single‐generation estimates of contemporary *N*_*e*_/*N*, which are the most informative for practical applications. It is crucial to clearly define which individuals are included in the census size (*N*). Defining *N* as the number of adults alive at a given time facilitates comparisons across species. For a wide range of applications and experimental designs, inbreeding *N*_*e*_ is simpler to calculate and interpret than variance *N*_*e*_. Effects of skewed sex ratio are generally modest, so most reductions to *N*_*e*_/*N* arise from overdispersed (greater‐than‐Poisson) variance in offspring number ($\sigma_{k}^{2}$). Even when fecundity changes with age, overdispersed within‐age variance generally contributes most to overall $\sigma_{k}^{2}$, and both random and deterministic (mediated by selection) factors can be important. Most species are age‐structured, so it is important to distinguish between effective size per generation (*N*_*e*_) and the effective number of breeders in one season or year (*N*_*b*_). Both *N*_*e*_ and *N*_*b*_ are important for applied conservation and management. For iteroparous species, a key metric is variance in lifetime reproductive success ($\sigma_{k•}^{2}$), which can be affected by a variety of additional factors, including variation in longevity, skip or intermittent breeding, and persistent individual differences in reproductive success. Additional factors that can be important for some species are also discussed, including mating systems, population structure, sex reversal, reproductive compensation, captive propagation, and delayed maturity.

## 1 INTRODUCTION

Effective population size (*N*_*e*_) is one of the most important parameters in evolutionary biology (Charlesworth, 2009), and it plays an increasingly vital role in applied conservation and management as well. Published exchanges regarding the “50/500” guideline (Frankham et al., 2014; Jamieson & Allendorf, 2012; Traill et al., 2010) have highlighted the importance of evolutionary considerations in endangered species management. 50 and 500 refer to *N*_*e*_ values believed to be necessary to maintain species viability in the short term and long term, respectively. This guideline or “rule‐of‐thumb” played an important role in shaping criteria developed by the International Union for Conservation of Nature (IUCN) that are widely used for identifying endangered species (IUCN, 2014; Mace et al., 2008; Mace & Lande, 1991). Some authors (e.g., Frankham et al., 2014) now argue that these guidelines are too low and should be doubled, to 100–1000. Recently, considerable efforts have been made to inject more meaningful genetic diversity criteria into the Convention on Biological Diversity's (CBD) post‐2020 global biodiversity framework (GBF), and *N*_*e*_ has been identified as a key metric to monitor (Hoban et al., 2021, 2022; Thurfjell et al., 2022).

Both in the scientific literature and within the conservation community, the ratio of effective size to census size has also generated a great deal of interest, for two main reasons. First, *N*_*e*_ is generally harder to estimate than *N*. If a generic *N*_*e*_/*N* ratio can be applied to a new species (for example, based on data for another species with similar life history), a rough estimate of *N*_*e*_ can be obtained from an estimate of the number of individuals in the population. Second, effective size is generally smaller than *N*, and the degree to which this is true provides important information about reproductive patterns and other aspects of the species' biology that can be useful in guiding conservation and management efforts. This information is particularly useful if the factors primarily responsible for reducing *N*_*e*_/*N* can be identified.

Here, I consider the practical applicability of the *N*_*e*_/*N* ratio to real‐world problems in conservation and management. The goal is not to review empirical estimates; meta‐analyses that were comprehensive for their time periods have been conducted by Frankham (1995), Palstra and Fraser (2012), and Clarke et al. (2024). Instead, emphasis will be on demographic and genetic methods for estimating effective size, census size, and their ratio. Considerable new work has been done in the last decade or so on how these estimates are affected by biological attributes (including age structure and spatial structure) that are commonly found in real populations.

This paper is organized as follows. Section 2 provides definitions for census size, effective size, and their ratio, including relevant time periods. This section also discusses the two major mechanisms by which effective size can be reduced compared to census size: a skewed sex ratio and greater‐than‐Poisson (overdispersed) variance in offspring number. Section 3 is a comprehensive look at the numerous attributes of real populations that can cause skewed sex ratios or overdispersed variance in reproductive success. Estimation of the *N*_*e*_/*N* ratio using demographic and/or genetic data is considered in Section 4. In Section 5, results from Sections 2, 4 are integrated to provide insights into theoretical and empirical ranges of *N*_*e*_/*N*. Finally, Section 6 reviews the major points and provides recommendations for practical implementation.

## 2 DEFINITIONS

### 2.1 Notation

Let *k*_*i*_ be the number of offspring individual *i* produces in one time period or season (hereafter assumed to be 1 year), and let *k•*_*i*_ = lifetime reproductive success (LRS) be the number of offspring that individual produces across its entire lifetime. Then, $u_{k}$ and $\sigma_{k}^{2}$ are the annual mean and variance of *k* for the whole population, respectively, and $u_{k•}$ and $\sigma_{k•}^{2}$ are the corresponding lifetime parameters. In age‐structured populations, age is typically indexed by *x*, and standard life tables typically use *b*_*x*_ to represent expected fecundity of individuals of age *x*. The associated age‐specific variance in fecundity is $\sigma_{k,x}^{2}$, and here we will also be concerned with the age‐specific variance‐to‐mean ratio, ${\phi_{x}=\sigma }_{k,x}^{2}/b_{x}$. Generation length (average age of parents of a newborn cohort) is calculated as $T=\frac{\sum xl_{x}b_{x}}{\sum l_{x}b_{x}}$ (Charlesworth, 1994), where *l*_*x*_ is cumulative survival through age *x*.

### 2.2 Census size

Because census size (*N*) is equally important as effective size in determining the *N*_*e*_/*N* ratio, it would be nice if *N* were consistently defined in the literature, but that is not universally the case. Whether immature individuals are included in the census size can profoundly affect the ratio, especially for species with delayed maturity and/or high juvenile mortality (see Section 5). Here, I follow Nunney and Elam (1994), Luikart et al. (2010), and others in defining *N* to be the adult census size = sum of numbers of adult males (*N*_*m*_) and adult females (*N*_*f*_). These represent sexually mature individuals alive at a given time, including those that might not breed that year or season. This definition should facilitate the most meaningful comparisons of *N*_*e*_/*N* ratios across diverse taxa.

### 2.3 Effective size

#### 2.3.1 Annual and generational effective sizes

Effective population size was originally defined for species having discrete generations (Wright, 1938). In subsequent applications to age‐structured species, the term *N*_*e*_ was used to refer to effective size over a period of one generation (Felsenstein, 1971; Hill, 1972, 1979; Johnson, 1977). Here, I follow this terminology and use *N*_*e*_ to refer to the generational effective size for a population. In age‐structured species, another important effective size can be identified: *N*_*b*_ = the effective number of breeders in 1 year or season (Waples & Teel, 1990). Although *N*_*e*_ per generation is more important in determining the rate of genetic drift, the *N*_*b*_/*N* ratio provides important information about mating systems and reproductive behavior. *N*_*b*_ also is generally much easier to estimate, both genetically and demographically. Demographic estimates of *N*_*e*_ require tracking individual reproductive success across the entire lifespan of the species, whereas each year or field season provides another opportunity to demographically estimate *N*_*b*_. Single‐cohort samples from 1 year of reproduction provide a robust way to genetically estimate *N*_*b*_, because the analytical framework more closely corresponds to the discrete‐generation assumption of the estimators. Furthermore, it is more straightforward to pair annual estimates of *N*_*b*_ with estimates of census size for the same time period than it is to properly match generational estimates of *N*_*e*_ and *N*. In the material below, both effective sizes are discussed.

#### 2.3.2 Flavors of *N*_*e*_

*N*_*e*_ controls the rates of both random allele frequency change and random increases in inbreeding. Effective size is often defined in terms of the rates of these evolutionary processes, but these definitions are not operational in any practical sense (Waples, 2022a). For application to real‐world problems in conservation and management, a demographic definition of effective size is much more useful. Crow (1954) was the first to define different effective sizes related to these two evolutionary processes. For a monoecious diploid with random self‐fertilization, the discrete‐generation inbreeding and variance effective sizes are as follows (Caballero, 1994):

(1) InbreedingNeI=ukNt−1−1uk+σk2uk−1,

(2) VarianceNeV=uk2Nt−121+σk2uk.

The subscript to *N* indicates the relevant generation of adult census size, with *t* being the offspring generation and *t*−1 the parental generation. In these equations, $u_{k}$ and $\sigma_{k}^{2}$ are calculated across the *N*_*t*−1_ parents that produced the sampled offspring. The two effective sizes are the same [*N*_*e*_ = (2*N*_*t*−1_ − 1)/(1 + $\sigma_{k}^{2}$/$u_{k}$)] only when population size is constant, such that $u_{k}=2$ (each parent providing half the genes to an average of two offspring). In general, variance *N*_*e*_ reflects reproduction in the offspring generation, while inbreeding reflects reproduction in the parental generation (Crow, 1954); as a consequence, *N*_*eV*_ is larger in increasing populations and *N*_*eI*_ is larger in declining ones.

*N*_*eI*_ is a more logical choice for computing the *N*_*e*_/*N* ratio, as then *N*_*e*_ in the numerator is computed for the same set of adults used in computing the census size in the denominator. With variance effective size, *N*_*eV*_ and *N* are offset by one generation. Furthermore, calculation of *N*_*eV*_ is very sensitive to the experimental design and sampling intensity. Because the sample variance in offspring number is positively correlated with the sample mean (Crow & Morton, 1955), variance *N*_*e*_ can be strongly upwardly biased if mean and variance in offspring number are measured from juvenile samples of a highly fecund species. In contrast, inbreeding *N*_*e*_ does not suffer from this problem and hence is much more useful for practical applications, especially in age‐structured species (Waples, 2002a, 2020).

It is also possible to define and estimate a long‐term or coalescent effective size, which reflects a balance between generation of genetic diversity by mutation and loss by genetic drift (Luikart et al., 2010; Wakeley & Sargsyan, 2009). This can provide useful deep‐time context for considering evolutionary processes in the focal species, but it is less relevant for applied conservation and management. Except as otherwise noted, the remainder of this paper focuses on inbreeding effective size of contemporary populations.

#### 2.3.3 Mechanisms that determine *N*_*e*_

##### Variance in offspring number

The importance of $\sigma_{k}^{2}$ and the variance‐to‐mean ratio $\phi =\sigma_{k}^{2}/u_{k}$ are apparent from Equation 1: if offspring number is Poisson distributed, such that ϕ=1, then

$$N_{\mathrm{eI}}\approx \frac{u_{k}N_{t-1}}{u_{k}}=N_{t-1},$$

as in an ideal Wright–Fisher population. More generally, if population size is constant (with $u_{k}=2$), then to a good approximation (Hedrick, 2000),

(3) $$\frac{N_{e}}{N}\approx \frac{2}{1+\phi }.$$

Equation 3 shows that the *N*_*e*_/*N* ratio depends heavily on ϕ, and *N*_*e*_/*N* is reduced to the extent that variance in offspring number exceeds the mean. Table 1 illustrates some hypothetical offspring distributions and resulting values of $\sigma_{k}^{2}$, ϕ, and *N*_*e*_/*N*.

**TABLE 1:** Hypothetical distributions of numbers of offspring per parent for 10 individuals and resulting values of inbreeding effective size (*N*_*eI*_from Equation 1) and *N*_*eI*_/*N*.

Scenario								
Individual	A	B	C	D	E	F	G	H
1	10	12	12	15	15	25	50	50
2	5	11	12	15	15	25	25	19
3	10	10	12	13	15	25	0	1
4	5	9	12	11	15	0	0	1
5	10	8	12	9	15	0	0	1
6	5	7	3	7	0	0	0	1
7	10	6	3	5	0	0	0	1
8	5	5	3	0	0	0	0	1
9	10	4	3	0	0	0	0	0
10	5	3	3	0	0	0	0	0
$u_{k}$	7.5	7.5	7.5	7.5	7.5	7.5	7.5	7.5
$\sigma_{k}^{2}$	6.3	8.3	20.3	33.3	56.3	131.3	256.3	230.5
ϕ	0.83	1.1	2.7	4.4	7.5	17.5	34.2	30.7
*N*_*e*_	10.1	9.7	8.0	6.8	5.3	3.1	1.8	2.0
*N*_*e*_/*N*	1.01	0.97	0.80	0.68	0.53	0.31	0.18	0.20

*Note*: In each scenario, mean number of offspring per parent is $u_{k}=7.5$, $\sigma_{k}^{2}$ is the population variance of *k*, and ${\phi =\sigma }_{k}^{2}/u_{k}$ is the variance‐to‐mean ratio.

##### Adult sex ratio

If the focal species has separate sexes, the adult sex ratio influences *N*_*e*_/*N*. If the sex ratio is equal and, within each sex, every individual is equally likely to produce offspring, then random mating will on average produce ϕ = 1 across both sexes and *N*_*e*_/*N* = 1 from Equation 3. If the numbers of adult males and females are not the same, each offspring still has one mother and one father, so individuals of the less‐numerous sex produce more offspring per capita. This increases the overall variance in offspring number (ϕ > 1 across both sexes) and reduces effective size. A skewed adult sex ratio can arise in two different ways: (1) a skewed primary sex ratio; (2) different survival rates in males and females. Most species have primary sex ratios close to 1:1, although there are some notable exceptions, especially for species with temperature‐sensitive sex determination (Janzen & Paukstis, 1991). Sex‐specific vital rates are found in many diverse taxa, and this factor commonly causes skewed adult sex ratios.

Wright (1938) showed that if variance in offspring number is Poisson within sexes, effective size is

(4) $$N_{e}=\frac{4N_{f}N_{m}}{N_{f}+N_{m}},$$

where *N*_*m*_ + *N*_*f*_ are numbers of males and females. As discussed later, the operational sex ratio can differ from that defined by the total numbers of each sex if some adults do not attempt to breed in a given year. Now *N*_*m*_ + *N*_*f*_ = *N*, so if we let *m* = *N*_*m*_/*N* and *f* = *N*_*f*_/*N*, then the *N*_*e*_/*N* ratio becomes

(5) $$\frac{N_{e}}{N}=\frac{4N_{f}N_{m}}{N^{2}}=4\frac{N_{f}}{N}\frac{N_{m}}{N}=4\mathrm{fm}.$$

**FIGURE 1:**
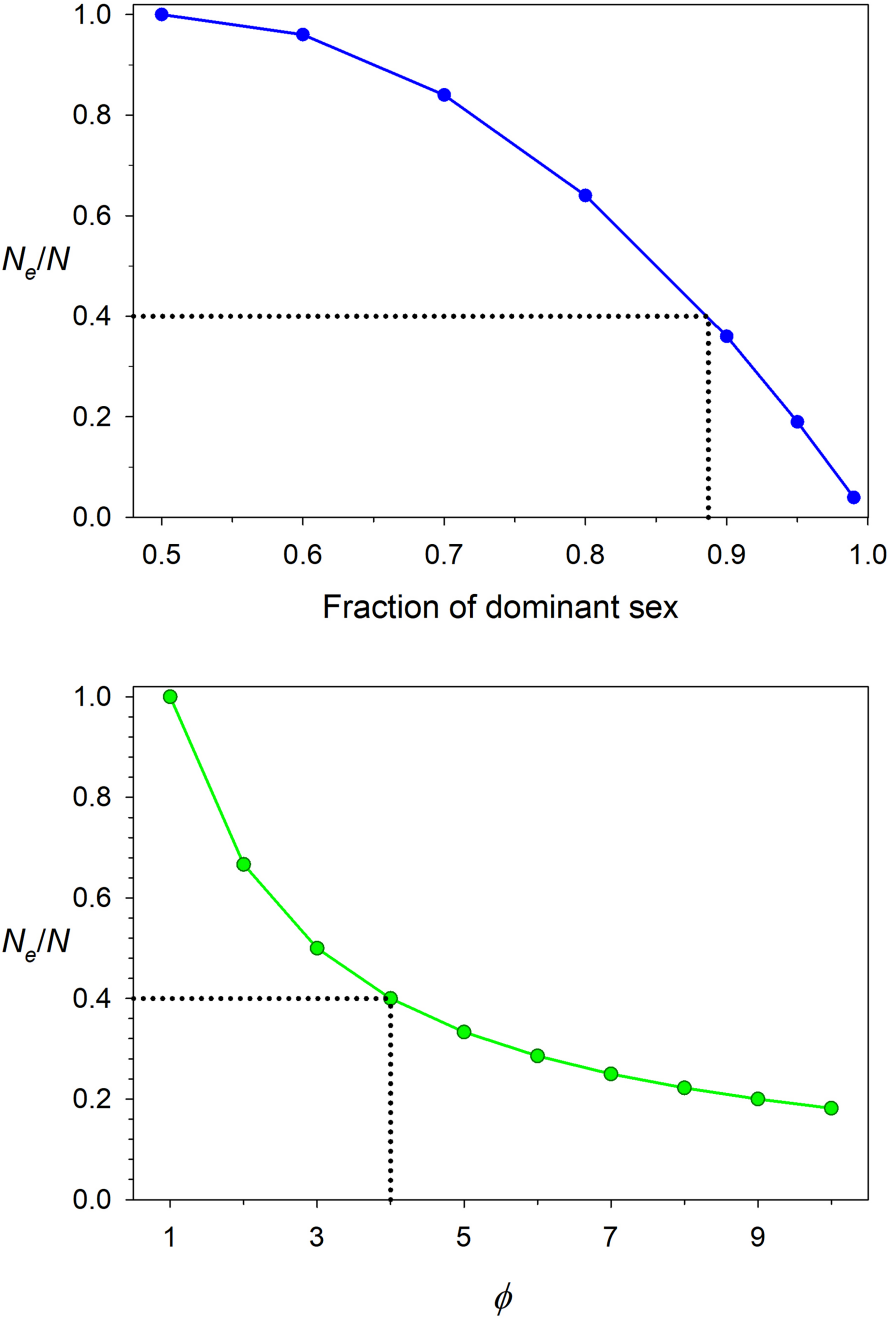
Effects of skewed sex ratio (top, based on Equation 5) and overdispersed variance in offspring number (bottom, based on Equation 3) on the *N*_*e*_/*N* ratio. Black dotted lines show that *N*_*e*_/*N* = 0.4 requires a nearly 9:1 adult sex ratio but can be achieved with ϕ=4 (variance in offspring number 4 times the mean).

It is easy to see from Equation 5 that the sex ratio must be strongly skewed to have an appreciable effect on *N*_*e*_. With *m*, *f* = 0.3, 0.7 (or vice versa—a greater than two‐fold disparity) *N*_*e*_/*N* = 0.84 – reduced by only 16%, and with a 9:1 sex ratio *N*_*e*_/*N* = 0.36 – still over a third of the value at sex‐ratio parity. Figure 1 illustrates the relative importance of skewed sex ratio (Equation 5) and overdispersed variance in reproductive success (Equation 3) in reducing *N*_*e*_/*N*.

For generalized applications, sex‐specific effective sizes ($N_{e\left(m\right)}$, $N_{e\left(f\right)}$) can be calculated and results used in Equation 4 to calculate the overall effective size:

(6) $$N_{e}\approx \frac{4N_{e\left(f\right)}N_{e\left(m\right)}}{N_{e\left(f\right)}+N_{e\left(m\right)}}.$$

As the ratio of the effective sizes becomes more extreme, the denominator converges on the larger effective size and overall *N*_*e*_ converges on a value equal to 4 times the smaller effective size.

### 2.4 The *N_e_*/*N* ratio

The final step in constructing the *N*_*e*_/*N* (or *N*_*b*_/*N*) ratio is to ensure that the numerator and denominator refer to the same time periods. Rules for doing this were provided by Waples (2005) and are discussed below. In theory, this step should be relatively straightforward, but Palstra and Fraser (2012) found that published estimates commonly create effective size: census size ratios that mix different time periods in the numerator and the denominator.

For applied conservation and management, the most relevant *N*_*e*_/*N* ratios are those that apply to single recent generations, as those estimates should be most predictive for what to expect in the future. If data for multiple previous generations are available, it is useful to consider the distribution of estimates for individual generations and whether those show a temporal trend or are correlated with *N* (see Section 5.2).

Some authors (e.g., Frankham, 1995; Vucetich et al., 1997) have claimed that fluctuations in abundance over time reduce the *N*_*e*_/*N* ratio. However, that is only true if one computes the multigenerational *N*_*e*_/*N* ratio as the harmonic mean *N*_*e*_ divided by the arithmetic mean *N* (Kalinowski & Waples, 2002; Waples, 2002b; see Table 2). Mixing harmonic and arithmetic means in a ratio creates a statistical artifact and guarantees that the multigenerational ratio will be smaller if there is any variation in *N*, even if single‐generational ratios do not vary. If one wants to compute a multigenerational *N*_*e*_/*N* ratio, a better approach is to compute it as either (1) (harmonic mean *N*_*e*_)/(harmonic mean *N*), or (2) the median or geometric mean of the single‐generation ratios (the arithmetic mean is not ideal as a measure of central tendency because ratios are seldom normally distributed) (Table 2).

**TABLE 2:** Calculation of multigeneration *N*_*e*_/*N*.

Generation	*N*	*N*_*e*_	*N*_*e*_/*N*	*N*	*N*_*e*_	*N*_*e*_/*N*	Generation	*N*	*N*_*e*_	*N*_*e*_/*N*
1	200	80	0.4	500	150	0.3	1	362	151	0.42
2	100	40	0.4	500	150	0.3	2	674	354	0.53
3	30	12	0.4	500	150	0.3	3	585	222	0.38
4	400	160	0.4	50	15	0.3	4	481	227	0.47
5	1000	400	0.4	500	150	0.3	5	460	270	0.59
Mean	346	138	0.4	410	123	0.3	6	602	145	0.24
Harmonic mean	96	39	0.4	179	54	0.3	7	681	343	0.50
${\tilde{N}}_{e}/\bar{N}$			0.11			0.13	8	523	164	0.31
Mean										0.43
Geomean										0.41
Median										0.44

*Note*: Left and middle columns: Hypothetical 5‐generation time series of *N*_*e*_ and *N* values, having a constant *N*_*e*_/*N* ratio of 0.4 (left) or 0.3 (middle). The multigeneration ratio is unchanged when calculated using either both arithmetic means (${\bar{N}}_{e}/\bar{N}$) or both harmonic means (${\tilde{N}}_{e}/\tilde{N}$), but it drops to 0.11 (left) or 0.13 (middle) if *N*_*e*_/*N* is calculated as harmonic mean *N*_*e*_ divided by arithmetic mean *N*. Right columns: Hypothetical 8‐generation time series of *N*_*e*_ and *N* estimates, leading to estimated *N*_*e*_/*N* ratios ranging from 0.24 to 0.59. Results of using three different measures of the central tendency of the *N*_*e*_/*N* estimates are shown.

## 3 FACTORS AFFECTING THE *N*_*e*_/*N* RATIO

### 3.1 Stochasticity

If newborns are equally likely to be male or female and subsequent survival probabilities are the same in both sexes, how much, on average, will *N*_*e*_ be reduced by random sex‐ratio differences? The answer is surprisingly simple: *N*_*e*_ will on average be reduced by exactly one individual (Waples & Do, 1994; Appendix). For tiny populations, this effect could be important, but if *N* is 100 or more the reduction to *N*_*e*_/*N* would be 1% or less. This result applies to discrete generations and to annual reproductive success in age‐structured species; for lifetime reproductive success in iteroparous species, it is also important to consider random variation in longevity, as discussed below.

Random processes are potentially more important in shaping variance in offspring number. As shown above, exactly implementing the Wright–Fisher model of random reproductive success leads to $E\left(\sigma_{k}^{2},/u_{k}\right)\approx 1$ and *N*_*e*_ ≈ *N*, so stochasticity on average reduces *N*_*e*_ by a factor of 2 from its theoretical maximum when $\sigma_{k}^{2}$ = 0. Two factors contribute to the realized value of $\sigma_{k}^{2}$: (1) production of offspring and (2) survival of offspring until the age at enumeration. In the Wright–Fisher model, these events are both *random* and *independent*. In particular, independence of survival means that whether individual X survives does not depend on whether individual Y or any other individuals also survive. But survival can be random and still not satisfy this independence criterion. For example, if X and Y are from the same family, they likely co‐occur in space and time during periods when substantial mortality occurs. As a consequence, siblings can experience family‐correlated mortality events from things like nest predation or simply (and collectively) being in the wrong place at the wrong time. Family‐correlated mortality increases $\sigma_{k}^{2}$ and reduces effective size compared to the Poisson expectation (Crow & Morton, 1955; Waples, 2002a). For a discussion of more generalized null models, see Kendall and Wittmann (2010) and Waples and Reed (2023).

### 3.2 Selection

Although family‐correlated mortality can be the result of bad luck, it also can be due to bad genes and hence mediated by natural selection. A simple way to model effects of selection on *N*_*e*_ and *N*_*e*_/*N* is to use a generalized Wright–Fisher model that allows for unequal parental probabilities of reproductive success (Waples, 2020, 2022b, building on earlier work by Robertson, 1961). These probabilities can be expressed as a vector of parental weights (***W***) that reflect the relative probability that an individual will be chosen as the parent of a given offspring. The parental weights are then equivalent to standardized selection coefficients. A convenient result is that a number of key population genetic indices are simple functions of the squared coefficient of variation of ***W***: ${\mathrm{CV}}_{W}^{2}=\sigma_{W}^{2}/{\bar{W}}^{2}$. For example, the expected value of the variance in reproductive success is

(7) $$E\left(\sigma_{k}^{2}\right)\approx \mu_{k}\left[1+\mu_{k}{\mathrm{CV}}_{W}^{2}\right],$$

and the expected *N*_*e*_/*N* ratio is

(8) $$E\left[\frac{N_{\mathrm{eI}}}{N}\right]\approx \frac{N}{1+{\mathrm{CV}}_{W}^{2}}.$$

Equation 8 is also valid for variance *N*_*e*_ but only in the special case of constant *N* (Felsenstein, 2019). If all the parental weights are equal, ${\mathrm{CV}}_{W}^{2}=0$ and Equations 7 and 8 reduce to $E\left(\sigma_{k}^{2}\right)=\mu_{k}$ and $E\left[N_{\mathrm{eI}}/N\right]=N$, as in the standard Wright–Fisher model. If ${\mathrm{CV}}_{W}^{2}$ is large, *N*_*e*_ can be reduced to a small fraction of census size.

### 3.3 Litter/clutch size constraints

Annual reproductive variance is constrained by the maximum number of offspring (*k*_max_) an individual can produce in 1 year/season. To the extent that such physiological constraints occur, they apply primarily to females, although male reproductive output also can be affected if the number of potential mates is limited. If the total number of offspring produced in the population in 1 year is *C* = Σ*k*_*i*_ and each female can produce no more than *k*_max_, this ensures that at least *C*/*k*_max_ females reproduce each year. If *k*_max_ is small, the random variance in female offspring number can also be constrained to be less than the Poisson variance, but this effect will generally be small unless *k*_max_ is <4 (Waples & Antao, 2014).

A special constraint occurs if litter size is small and fixed at *D*. Apart from rare multiple births, females of many large mammals produce no more than one offspring annually, and many long‐lived seabirds produce only a single egg annually, while many penguin species (Borboroglu & Boersma, 2012) and the gecko *Oedura reticulata* (Hoehn et al., 2012) always lay exactly two eggs. If the only options for a female each year are to produce 0 or *D* offspring, and if *X* of *N*_*f*_ females successfully reproduce, then the annual female variance in offspring number is as follows (Waples & Antao, 2014):

(9) $$\sigma_{k\left(f\right)}^{2}=\mu_{k\left(f\right)}D\left[1-\frac{X}{N_{f}}\right].$$

Mean offspring number for females is $\mu_{k\left(f\right)}=\mathrm{DX}/N_{f}$, which is also the Poisson variance, so the term $D\left[1-X/N_{f}\right]$ represents the degree to which $\sigma_{k\left(f\right)}^{2}$ is reduced from the random expectation.The resulting female inbreeding effective size is obtained by inserting the result from Equation 9 into Equation 1, leading to

(10) $$N_{\mathrm{bI}\left(f\right)}=\frac{\mathrm{XD}-1}{D-1}.$$

For *D* = 1, annual $N_{\mathrm{bI}\left(f\right)}$ is infinitely large, reflecting the fact that, because each offspring has a different mother, no increase in female inbreeding can occur within a single year. In that case, the overall annual effective size is 4 times male $N_{\mathrm{bI}\left(m\right)}$.

### 3.4 Age structure

**FIGURE 2:**
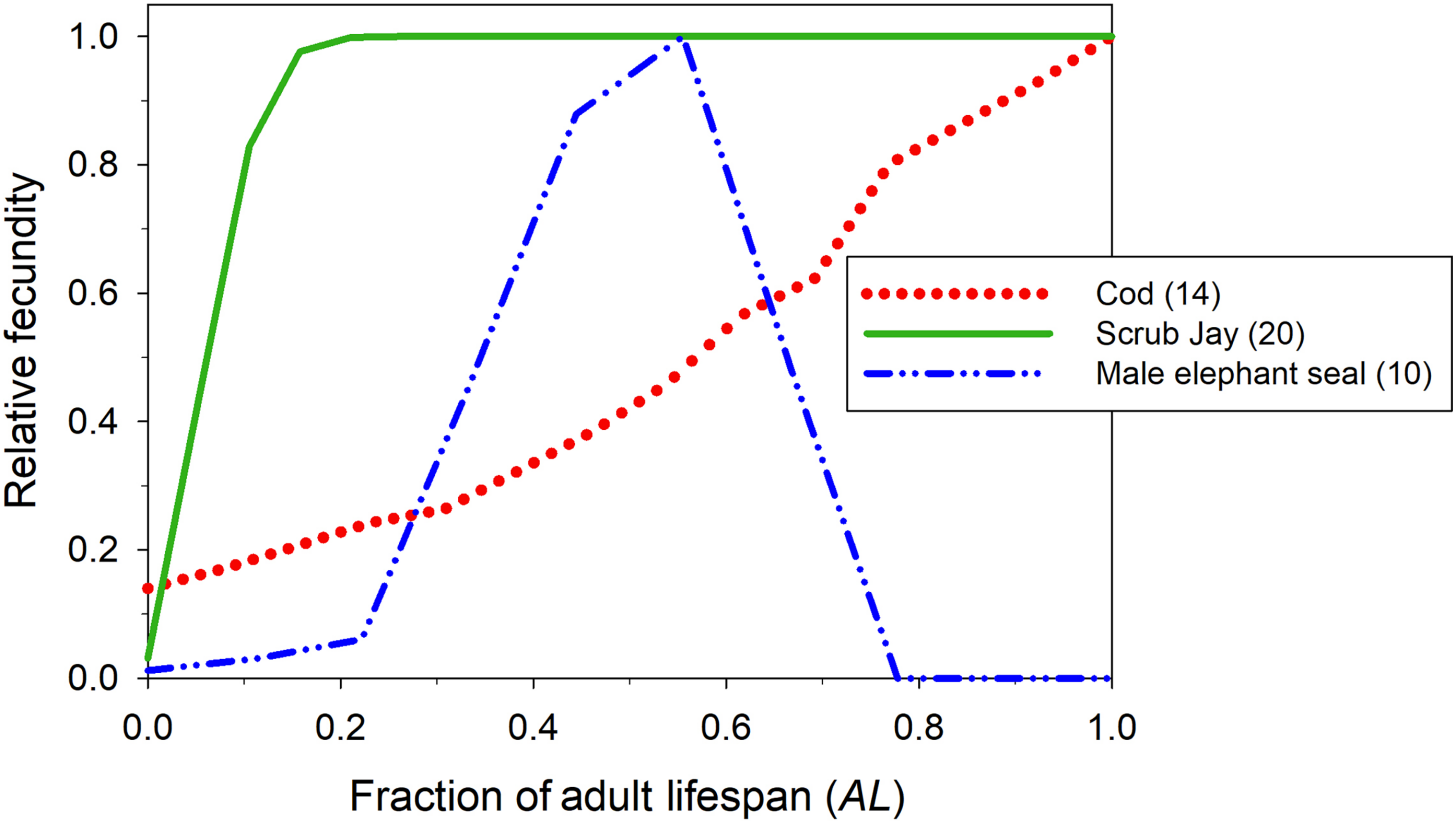
Patterns of change in expected fecundity with age in three species. The *X* axis is scaled to the adult lifespan of each species, defined as *AL* = *ω* – *α* + 1, where *α* = age at first reproduction and *ω* = maximum age. On the *Y* axis, age‐specific fecundity (*b*_*x*_ = mean number of offspring produced by individuals of age *x*) is scaled as a fraction of the maximum for each species. Constant fecundity after maturity is common in birds, including the Florida scrub jay (*Aphelocoma coerulescens*; Woolfenden & Fitzpatrick, 1984) shown here. Many species, especially ectotherms with indeterminant growth like Atlantic cod (*Gadus morhua*; Hutchings, 2005), have increasing fecundity that is roughly proportional to age. The male elephant seal (*Mirounga angustrirostris*, LeBoeuf & Reiter, 1988) shows a hump‐shaped pattern typical of species with harem polygamy. Numbers after species names are adult lifespan in years.

By itself, age structure does not affect annual $\sigma_{k}^{2}$ or *N*_*b*_, which are calculated across all mature individuals alive in a given year. However, when expected fecundity (*b*_*x*_) varies by age (Figure 2), overall $\sigma_{k}^{2}$ will be greater than the mean even if reproductive variance is Poisson within ages. It is therefore useful to partition the overall annual variance into within‐age and between‐age components, which can be done using a one‐way ANOVA sums‐of‐squares approach (Waples, 2023a). The between‐age component depends on the pattern of change in fecundity with age, and the within‐age component depends on the variance‐to‐mean ratio within each age, $\phi_{x}$. If all *b*_*x*_ = *b* and all $\phi_{x}=1$, then all adults behave like an ideal population each year, with *E*(*N*_*b*_) = *N*.

**FIGURE 3:**
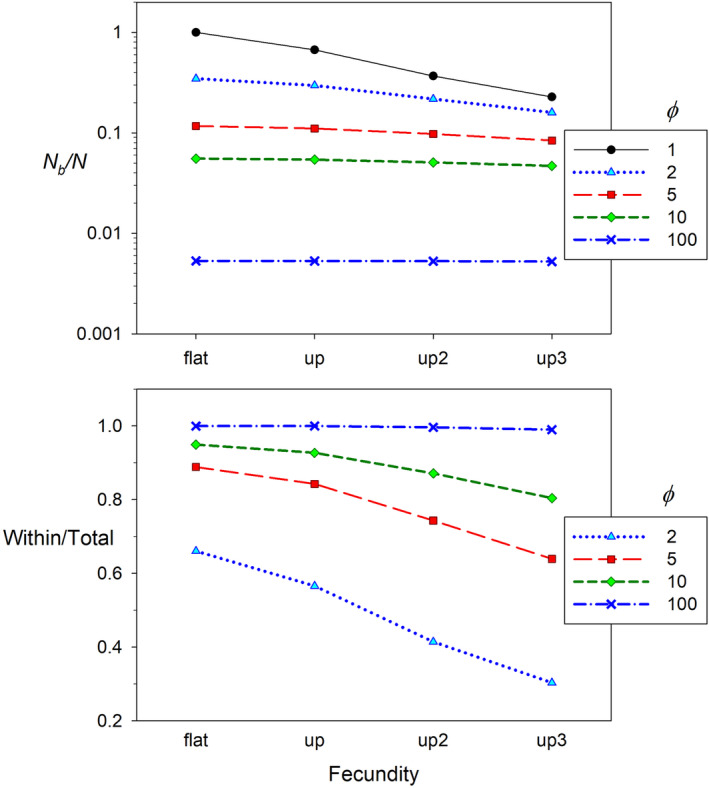
Effects of patterns of age‐specific fecundity and within‐age variance in offspring number. The four fecundity patterns shown on the *X* axis are “flat” (constant fecundity with age), “up” (fecundity proportional to age), “up2” (fecundity proportional to age^2^), and “up3” (fecundity proportional to age^3^). In each fecundity scenario, individuals of all ages had variance in offspring number a multiple of the age‐specific *b*_*x*_ values, as indexed by ${\phi_{x}=\sigma }_{k,x}^{2}/b_{x}$ ranging from 1 to 100. Top: *Y* axis (note the log scale) shows the *N*_*b*_/*N* ratio. Bottom: *Y* axis shows the fraction of the overall reduction from *N*_*b*_/*N* = 1 that is due to overdispersed within‐age variance. With all $\phi_{x}=1$, there is no within‐age contribution to reduction in *N*_*b*_/*N* below the Poisson expectation of 1, so results are only shown for $\phi_{x}\geq 2$. Before the analysis, *b*_*x*_ was rescaled to values that will produce a stable population, and the $\phi_{x}$ values shown reflect the rescaled variance‐to‐mean ratios. Age‐specific vital rates used for this figure are in Table S1.

Figure 3 shows results for simulated scenarios with 10 adult age classes, where *b*_*x*_ was either invariant or was proportional to age, age^2^, or age^3^, and *ϕ* ranged from 1 to 100. With only Poisson variation in offspring number within ages (all $\phi_{x}=1$), *N*_*b*_/*N* was only reduced to ~0.3 even for the most extreme fecundity scenario (in which expected fecundity at age 10 was 1000 times that for an age‐1 individual; Figure 3a). In contrast, even when fecundity was invariant with age, *N*_*b*_ was only ~10% of *N* with $\phi_{x}=5$ at each age, and the *N*_*b*_/*N* ratio can be arbitrarily small as *ϕ* becomes arbitrarily large. Except under scenarios where fecundity increased sharply with age and $\phi_{x}$ was low (≤2), most of the reduction in *N*_*b*_/*N* was due to overdispersed variance among individuals of the same age and sex (Figure 3b). Note that although in these simulations $\phi_{x}$ was always the same for every age, that need not be the case in real populations (unpublished data). For example, *ϕ* increases with age in Atlantic cod (Kuparinen et al., 2016) and male black bears (Waples, Scribner, et al., 2018).

In semelparous species with variable age at maturity, an individual's annual reproductive success is also its lifetime reproductive output. As a consequence, generational *N*_*e*_ is a simple function of *T* and the arithmetic mean (${\bar{N}}_{b}$) or harmonic mean (${\tilde{N}}_{b}$) effective number of breeders in the years comprising a generation:

(11) $$N_{e}=T{\bar{N}}_{b}$$

annual plants with seedbanks; Nunney (2002);

(12) $$N_{e}=T\tilde{N}$$

Pacific salmon and monocarpic perennials; Waples (2002c, 2006).

As discussed by Waples (2006), the arithmetic/harmonic mean difference arises because of different methods of population regulation in the two groups of species. For these species, the distribution of recent *N*_*b*_/*N* estimates provides the most reliable insights into what to expect in the future. If one wants a generational census size to pair with the *N*_*e*_ values in Equations 11 and 12, one can follow Nunney (2002) and Waples et al. (2010) and use the sum of all the different mature adults across a generation (*N*_*T*_).

In iteroparous species, *N*_*e*_ per generation depends on both generation length and the variance in LRS. The most widely used formula for *N*_*e*_ in species with overlapping generations is by Hill (1972, 1979). If the population has stable age structure and produces a constant number (*N*_*1*_) of offspring in each cohort, generational effective size is given by

(13) $$N_{e}\approx \frac{4N_{1}T}{\sigma_{k•}^{2}+2},$$

where $\sigma_{k•}^{2}$ is calculated for the *N*_*1*_ individuals in a cohort. As with annual reproduction, if vital rates differ between the sexes, it is best to compute separate effective sizes for males and females and combine them using Equation 6.

Within each sex, the lifetime variance $\sigma_{k•}^{2}$ is affected by the age‐specific parameters *b*_*x*_ and $\phi_{x}$, as well as two factors that are not involved in annual reproduction: (1) variation in longevity, and (2) temporal correlations in individual reproductive success. Variation in longevity is inevitable as, by pluck or luck (Snyder & Ellner, 2018), some individuals live longer than others and have more opportunities to add to their LRS. If fecundity increases with age, as is common in many species, the benefits of living longer are magnified, and $\sigma_{k•}^{2}$ is correspondingly increased.

Temporal autocorrelations in reproductive success can be either positive or negative, and the degree to which they occur varies widely across taxa. Temporary negative correlations arise from the phenomenon known as skip or intermittent breeding, which is common in many species, especially females (Bull & Shine, 1979; Goutte et al., 2011; Prince & Chaloupka, 2012). This life history strategy generally reflects the physiological/energetic costs of reproduction and can be quantified by the vector *θ*, where *θ*_*i*_ is the probability that an individual will reproduce in the current year, given that it last reproduced *i* years ago (Shaw & Levin, 2013). Skip breeding has a relatively large effect on *N*_*e*_/*N* in species with Type III survivorship (high juvenile mortality) (Waples & Antao, 2014). Permanent negative correlations arise from trade‐offs between reproduction and survival, such that reproduction in a given year reduces an individual's probability of survival. Negative temporal autocorrelations in reproductive success reduce $\sigma_{k•}^{2}$ (and hence increase *N*_*e*_) because they ensure that the same individuals cannot dominate reproduction every year.

Positive autocorrelations (aka persistent individual differences; Lee et al., 2011) occur when some individuals are consistently better or worse than average (for their age and sex) at producing offspring. Some life histories are a priori more likely than others to generate persistent differences. For example, in ectotherms with indeterminate growth (most fishes, some reptiles and amphibians, and many invertebrates), expected fecundity is typically proportional to size, and an individual that is large for its age in  a given year is likely to also be large for its age in subsequent years. Positive autocorrelations increase disparities among individuals in LRS and hence increase $\sigma_{k•}^{2}$ and reduce *N*_*e*_ and *N*_*e*_/*N*. Waples (2023b) introduced a new metric (ρ_*α*,__*α*+_) to quantify the magnitude (if any) of temporal autocorrelations. ρ_*α*,__*α*+_ is the correlation between (a) the number of offspring produced by each individual at its first age at reproduction, and (b) the total number of offspring produced during the rest of that individual's lifetime. As data accumulate for more species, it might be possible to establish empirical relationships between ρ_*α*,__*α*+_ and $\sigma_{k•}^{2}$.

### 3.5 Mating systems

The original Wright–Fisher model assumed a monoecious diploid with random selfing. Differences in *N*_*e*_ between this system and otherwise ideal populations with separate sexes and either random mating or lifetime monogamy are negligible (Felsenstein, 2019). Mating systems that exclude a fraction of adults from reproduction do have the potential to lower *N*_*e*_/*N*. As female offspring production determines the size of the next generation, restrictions on female reproduction cannot be too severe if the population is to remain stable—except perhaps in species with very high fecundity (Hedgecock & Pudovkin, 2011). Males do not have the same constraint, and male polygamy can sharply reduce annual *N*_*b*_/*N* and potentially generational *N*_*e*_/*N*.

In northern elephant seals, at any given time, a few dominant males monopolize virtually all reproduction, with young males too young/small to secure a harem, and older, over‐the‐hill males shunted to the sidelines. Males mature no earlier than age 6 and on average their reproductive output peaks at ages 10–11 before rapidly dropping to zero (Figure 2). For any given year, this means that male *N*_*b*_ is much lower than the number of adult males. However, the extent of this reduction, and the consequences for generational *N*_*e*_/*N*, cannot be determined without two additional key pieces of information. First, what is the variance in reproductive success among the males aged 10–11 years? Do they all have harems, in which case $\phi_{x}$ for those years might be close to 1? Or do only some have harems, in which case $\phi_{x}$ could be very large? The answer to this question most strongly affects *N*_*b*_/*N*, but it also is relevant to *N*_*e*_/*N*. Second, how long does a typical male hold a harem? If it is only for a year or two, that would mean many more males eventually have a chance to produce offspring, which would limit the magnitude of the reduction in *N*_*e*_/*N*. But if at least some males can hold harems for many years, persistent individual differences would increase the lifetime variance $\sigma_{k•}^{2}$ and reduce *N*_*e*_/*N*.

### 3.6 Migration and population structure

All of the above material assumes a single closed population. Most real populations are connected to others by some level of migration and hence are part of a metapopulation. As a consequence, it is important to consider effectives sizes of both the local population and the metapopulation as a whole. In Wright's (1943) island model, metapopulation *N*_*e*_ is greater than the sum of the effective sizes of the local populations, but that occurs only because of unrealistic model assumptions – in particular, that subpopulations are fixed in size and hence immortal. Under arguably more realistic scenarios that allow for subpopulation extinction/recolonization and/or variation in contributions to the migrant pool, metapopulation *N*_*e*_ can be less than the sum of the local *N*_*e*_s (Hedrick & Gilpin, 1997; Nunney, 1999; Whitlock & Barton, 1997).

**FIGURE 4:**
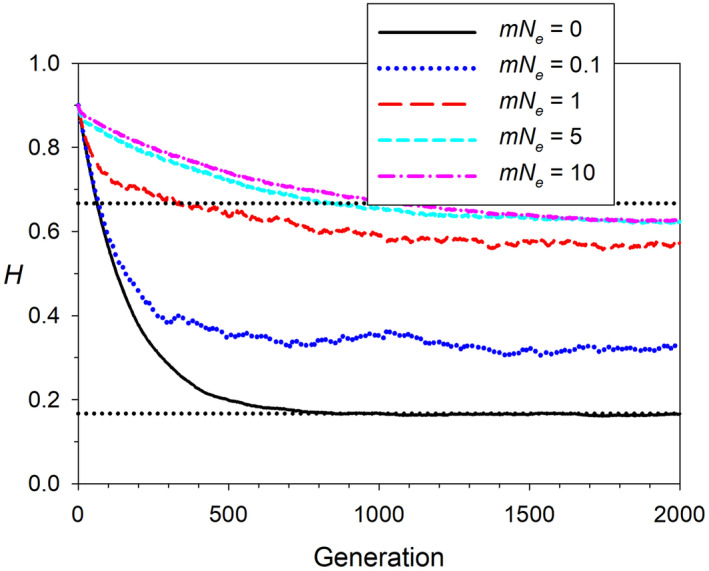
Mean heterozygosity (*H*) in simulated local populations experiencing various levels of gene flow (indexed by *mN*_*e*_ = effective number of migrants per generation) from a metapopulation following Wright's island model of migration. Local *N*_*e*_ was 100 for each of 10 subpopulations, and heterozygosity was tracked at 1000 “microsat” loci initially having 10 equally‐frequent alleles each. After 1000 or more generations, local *H* eventually reached an equilibrium between loss of alleles by drift and generation of new diversity arising from a mutation rate of *μ* = 5 × 10^−4^. At equilibrium in a closed population, *E*(*H*) = *θ*/(*θ* + 1), where *θ* = 4*N*_*e*_ *μ*. For an isolated local population, *θ* = 4 × 100 × 5 × 10^−4^ = 0.2, so the asymptotic *H* in the simulations (~0.165) is in good agreement with the theoretical expectation of 0.2/1.2 = 0.167 (lower dotted black line). With a high migration rate of *mN*_*e*_ = 5 or 10, asymptotic local *H* was 0.622 and 0.626, respectively—not far below the expected value (0.667; upper black dotted line) for a single panmictic population with overall *N*_*e*_ = 1000 and *θ* = 2. Data were simulated in EasyPop (Balloux, 2001).

Not all evolutionary processes are affected in the same way by metapopulation dynamics. Consider the common scenario where one is primarily concerned with evolutionary behavior within a local population, and all sampling is local. If the population is demographically independent (such that the population trajectory is driven more by local birth and death rates than by immigration/emigration), then the rates of allele frequency change and increase in inbreeding, the amount of random LD generated, and mating‐system dynamics will not, in general, be strongly affected by metapopulation processes (Gilbert & Whitlock, 2015; Luikart et al., 2010; Waples & England, 2011). In contrast, the amount of genetic diversity within a local population is strongly influenced by even very small amounts of migration (Ryman et al., 2019; Waples, 2010). For example, in an isolated population with constant *N*_*e*_ = 100, under a *k*‐allele mutation model with mutation rate of 5 × 10^−4^ (typical for microsatellites), equilibrium heterozygosity is *H* ≈ 0.167 (Figure 4). But if this local population is connected to 9 others by island model migration, heterozygosity in the local population soars to ~0.56 with a single migrant per generation, and with only one migrant every 10 generations, local *H* is twice as high as under complete isolation. Local *N*_*e*_ is therefore not a good indication of the amount of local genetic diversity, except under essentially complete, long‐term isolation. With respect to the 50/500 rule, this suggests that the *N*_*e*_ ≥ 500 criterion should be evaluated with respect to metapopulation *N*_*e*_, not local *N*_*e*_.

### 3.7 Sex reversal

Sex reversal (sequential hermaphroditism) is found in over a dozen invertebrate phyla and within at least 27 families of fishes, with both protogyny (female first, changing to male) and protandry (male→female) being widespread (Benvenuto et al., 2017; Policansky, 1982). These species generally have sex ratios strongly skewed toward the primary sex, which suggests that they might have low *N*_*e*_/*N* ratios (cf Equation 5). However, calculating LRS in these species requires integration of lifetime reproductive output across both sexes. It turns out that, if a species changes sex at the evolutionarily stable strategy (ESS) age for sex reversal (Charnov, 1993), *N*_*e*_/*N* is generally equal to or slightly larger than would be found in a gonochoristic species having the same vital rates and an equal sex ratio (Waples, Mariani, & Benvenuto, 2018). Thus, sex reversal allows these species to effectively avoid the evolutionary cost of skewed sex ratios experienced by gonochoristic species.

### 3.8 Captive propagation

Captive propagation can profoundly affect *N*_*e*_ and *N*_*e*_/*N* in either direction, depending on the circumstances. Consider first a scenario involving a single population, with all reproduction occurring in captivity. Assume as an example that 10 adult females and 15 adult males are available for breeding. One might be tempted to equalize the number of breeding males and females, since a skewed sex ratio reduces *N*_*e*_/*N*. However, unless additional females can be found, equalizing the sex ratio would require not using some males, and that would reduce *N*_*e*_ even though *N*_*e*_/*N* increased [using all adults and assuming Poisson variance in reproductive success within sexes, *N*_*e*_ = 4 × 10 × 15/(10 + 15) = 600/25 = 24, compared to *N*_*e*_ = 4 × 10 × 10/(10 + 10) = 20 if sex ratio of breeders is equalized]. A more effective strategy for increasing *N*_*e*_ in captivity is to reduce $\sigma_{k}^{2}$. One goal of captive population is to provide a protective environment that avoids much of the mortality that occurs in the wild. For species with high juvenile mortality, including most fishes, this can potentially provide an order‐of‐magnitude increase in survival. This in turn provides opportunities for culling eggs or offspring to equalize family sizes, which reduces $\sigma_{k}^{2}$ and increases effective size (Allendorf, 1993; Lind et al., 2010). A caveat is that for some species, equalization is most easily done at very early life stages, and subsequent uncontrolled mortality can limit the benefits to *N*_*e*_ and *N*_*e*_/*N*.

**FIGURE 5:**
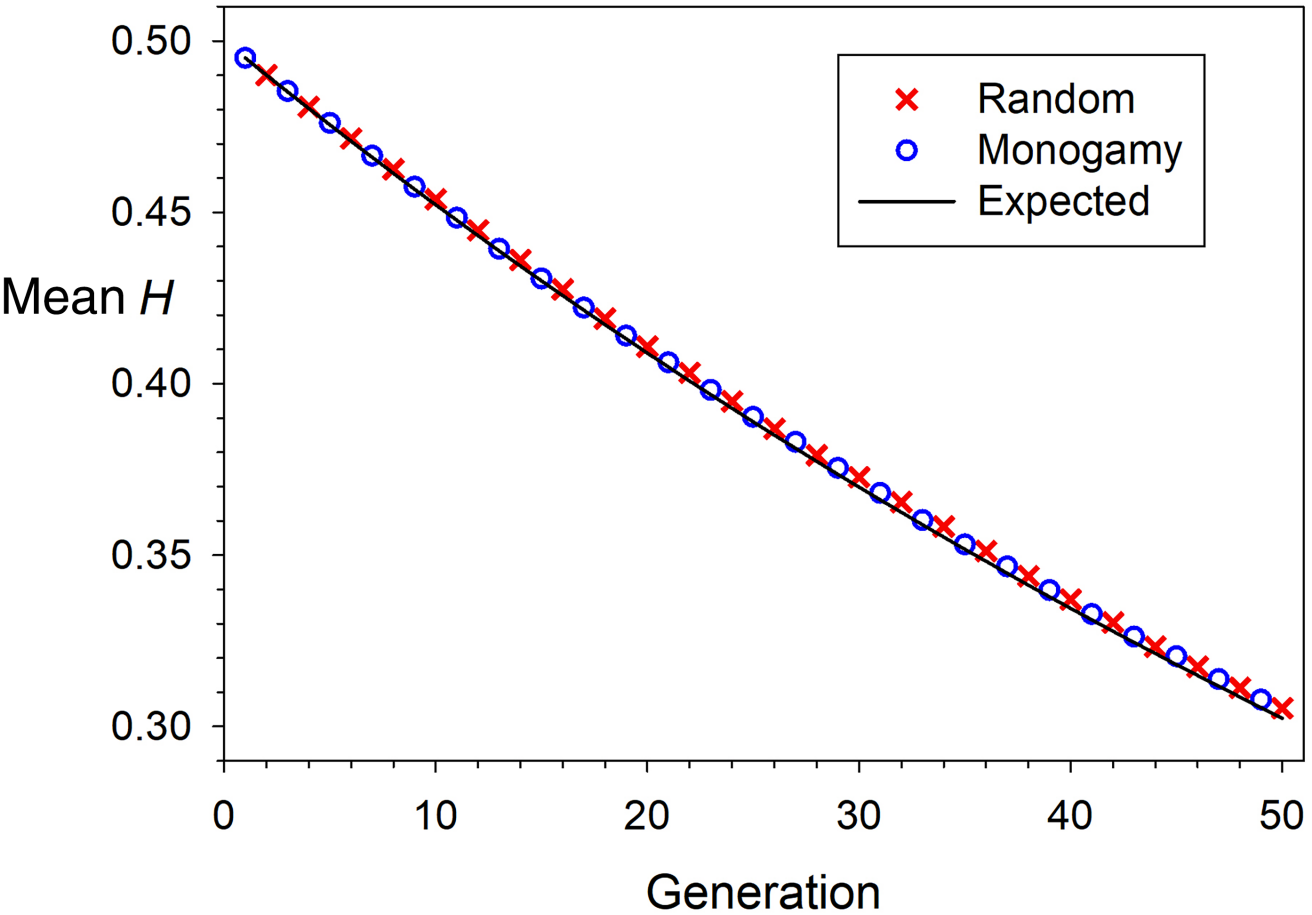
Empirical rates of decline in expected heterozygosity (*H*) in simulated populations with 25 males and 25 females, all with equal expected reproductive success. Red Xs are for a Wright–Fisher model of random mating of males and females independently to produce each offspring; blue circles are for a monogamy model, where each female is permanently paired with a different male, and each offspring is produced by randomly choosing a pair of parents. The solid black line is the theoretically expected value of *H* at generation *t*, according to the formula *H*_*t*_ = *H*_*0*_[1–1/(2*N*_*e*_)]^*t*^ (Crow & Kimura, 1970), assuming no mutation, *N*_*e*_ = 50, and initial *H* = 0.5 at generation 0. Results shown are averages across 50 replicate runs of 50 generations, each tracking frequencies of 500 diallelic loci.

Another strategy commonly used with fecund species is to allow each female to mate with several males. Compared to strictly pairwise matings, this creates more families and a more diverse array of genotypes in the offspring, which in turn provides more raw material for natural selection to act upon. If creating multiple families per female helps equalize offspring number, it also can increase *N*_*e*_ as described above. By itself, however, increasing the number of mates has little effect on *N*_*e*_ or *N*_*e*_/*N* (see Figure 5), with the primary benefit being that a sterile individual would not also nullify all the genetic contributions of another individual (its mate), but would instead reduce somewhat the reproductive output of several mates.

Managers of captive populations often use genetic markers or pedigree information to avoid matings between close relatives (Ballou & Lacy, 1995; Caballero et al., 1996; Doyle et al., 2001). This can have short‐term benefits by reducing the incidence of homozygosity for deleterious alleles—and hence expression of inbreeding depression—but it is not an effective long‐term strategy for increasing *N*_*e*_ or *N*_*e*_/*N*. In a small, closed population, even “unrelated” individuals share many alleles that are recently identical by descent, and very quickly, these combine within individuals to produce levels of IBD that are not substantially different from expectations under random mating. The problem is analogous to that faced by a juggler trying to keep multiple balls in the air. A talented juggler might keep a few balls in play for a while, but if she attempts too many, most will end up on the ground (see Allendorf et al., 2022 p. 499 for related discussion of inbreeding).

Effects of captive propagation are more complicated in scenarios where only part of a population is propagated in captivity, and the captive and wild components regularly exchange genes. Such programs were referred to as “supportive breeding” by Ryman and Laikre (1991) and are carried out on a gigantic scale globally for a wide range of taxa (Laikre et al., 2010). In these programs, *N*_*e*_ for the captive‐wild system as a whole depends on (1) the fraction of the population in captivity, (2) *N*_*e*_ or the *N*_*e*_/*N* ratio in both captivity and the wild, (3) duration of the captive program, and (4) patterns of post‐supplementation abundance (Lynch & O'Hely, 2001; Ryman & Laikre, 1991; Waples et al., 2016; Waples & Do, 1994). With so many variables involved, the range of potential outcomes is large. In general, however, any captive program that makes an appreciable genetic contribution to the wild population will almost always reduce overall *N*_*e*_ (compared to wild *N*_*e*_ in the absence of a program) unless *N*_*e*_/*N* in captivity is much higher than it is in the wild (Waples et al., 2016).

### 3.9 Exploitation

Exploitation of a natural population increases adult mortality, and this has predictable consequences for effective size. Reduced survival means that fewer individuals live to older ages, which directly reduces adult *N* and indirectly reduces *N*_*e*_. However, reductions to *N*_*e*_ are not as extreme as reductions to *N*, so the *N*_*e*_/*N* ratio actually increases (Kuparinen et al., 2016). The reason for this is that truncating the adult lifespan reduces the opportunity for long‐lived individuals to accumulate very high LRS. This in turn reduces $\sigma_{k•}^{2}$ and leads to higher *N*_*e*_/*N*, even as *N*_*e*_ declines. This phenomenon is particularly strong in species for which fecundity increases with age. It does not require that exploitation be size‐selective; simply increasing overall adult mortality will produce this kind of result. However, the effect is magnified if larger/older individuals are removed at a higher rate. Similarly, this effect does not require fishery‐induced evolution (FIE; Kuparinen & Merilä, 2007; Heino et al., 2015), which creates evolutionary pressure to mature earlier and at a smaller size; again, however, effects are magnified if this occurs.

In addition to human exploitation, natural phenomena can increase adult mortality and lead to comparable results. For example, climate change is expected to make global oceans warmer and more acidic, with less dissolved oxygen (Hoegh‐Guldberg et al., 2014). This will increase adult mortality and create evolutionary pressure for faster marine life histories, just as FIE does (Waples & Audzijonyte, 2016). A major difference is that whereas consequences of FIE are confined to the relatively few species harvested by humans, warming oceans can be expected to reduce *N*_*e*_ but increase *N*_*e*_/*N* for vast numbers of marine ectotherms.

## 4 ESTIMATION

### 4.1 Census size

A wide variety of methods can be used to estimate abundance of natural populations (Borchers et al., 2002; Seber, 1986). One that has been in common use for over half a century is mark–recapture (Jolly, 1965; Seber, 1982), which in its simplest form involves three steps: (1) capture a sample of *S* individuals; (2) mark and then release them to mingle with their peers; (3) collect another sample and record the fraction (*P*) that are marked; these marked individuals are the “recoveries.” A simplistic estimate of abundance is then $\hat{N}=S/P$. In practice, a wide range of covariates (including tag loss, mortality, movement in or out of the study area, and tag‐induced behavior affecting recapture) must be accounted for to obtain a robust estimate. Luikart et al. (2010) reviewed use of DNA markers to estimate *N* in mark–recapture and rarefaction studies.

A variation of this method that recently has gained considerable traction is close‐kin mark–recapture (CKMR; Bravington, Grewe, et al., 2016 Bravington, Skaug, et al. 2016). Taking advantage of the fact that close relatives naturally share genes (“marks”), CKMR obviates the need to sample any individual more than once. The two categories of close kin that have been used to date are parent–offspring pairs (POPs) and siblings. Estimation is conceptually similar to mark–recapture: the numerator of the simplified version of $\hat{N}$ is the number of pairwise comparisons of individuals to search for close‐kin matches, and the number of matches (aka “recoveries”) is in the denominator. As with traditional mark–recapture, precision of CKMR estimates depends on the number of recoveries (*R*), such that an approximate minimum CV to $\hat{N}$ is $1/\sqrt{R}$ (Bravington, Grewe, et al., 2016). As also is the case with traditional mark–recapture, a variety of covariates must be considered, and estimation is best done in an integrated likelihood framework where all key parameters can be jointly estimated. Early implementations of CKMR used POPs (Bravington, Grewe, et al., 2016), but with improved genomics technology, it is now feasible to assay thousands of SNPs to reliably identify pairs of siblings. Whereas incidence of siblings within cohorts provides information related to effective size (Wang, 2009), cross‐cohort siblings give insights regarding adult abundance (Waples & Feutry, 2022). Akita (2020) suggests using mtDNA to estimate the female *N*_*e*_/*N* ratio by jointly estimating female *N* using maternal POPs and female *N*_*e*_ using maternal siblings born in the same year.

### 4.2 Effective size

Effective size can be estimated using demographic or genetic methods. As each has its own strengths and weaknesses, the most robust estimates often come from a combination of approaches. In general, effective size is best measured across a full life cycle. Quantitative geneticists prefer a zygote‐to‐zygote experimental design because that avoids mixing signals of reproduction by parents and survival of their offspring (Thomson & Hadfield, 2017). However, this predictably leads to zero‐inflation of $\sigma_{k}^{2}$ due to inclusion of (potentially large numbers of) individuals that do not survive to reproductive age (Waples & Reed, 2023). A more meaningful framework for considering *N*_*e*_ and the *N*_*e*_/*N* ratio is to consider production of adults by adults. In many cases, it is easier to sample offspring before they reach sexual maturity. This does not present any analytical problems for estimating inbreeding effective size, but it should be remembered that in that scenario the estimates apply to only part of a generation.

#### 4.2.1 *N*_*b*_ per year

##### Demographic estimates

Modern genomics tools now make it relatively easy to obtain demographic estimates of *N*_*b*_ from parentage analysis, provided adequate samples can be collected. Parentage analysis provides a vector of numbers of offspring per parent, from which it is easy to compute the mean and variance in offspring number and *N*_*bI*_ using Equation 1. Partial sampling of offspring does not lead to bias, provided sampling is random with respect to family. This can be difficult to achieve, as naïve sampling methods might collect siblings at higher frequencies than they would occur at random. Many researchers purge putative siblings before conducting downstream analyses, but this can cause more problems than it solves and never is a good idea when one wants to estimate *N*_*e*_ or *N*_*b*_ (Waples & Anderson, 2017). If only a fraction of parents are randomly sampled and available for matching to offspring, ${\hat{N}}_{b}$ will be downwardly biased, but the estimate of *N*_*b*_/*N* will not be.

A demographic estimate of *N*_*b*_ can also be obtained from age‐specific data in a standard life table (survival and fecundity), provided that information is also available on the ratio of the variance‐to‐mean offspring number for individuals of the same age and sex (*ϕ*). The software AgeNe (Waples et al., 2011) makes these computations.

##### Genetic estimates

Estimating *N*_*b*_ indirectly using genetic markers also is straightforward, given appropriate samples (Luikart et al., 2010). The single‐sample methods based on LD (Waples & Do, 2008) and siblings (Wang, 2009) both estimate inbreeding *N*_*e*_ when applied to species with discrete generations, and both estimate inbreeding *N*_*b*_ for single‐cohort samples from age‐structured species. Extensive evaluations (Wang, 2016; Waples & Do, 2010) show that precision is generally high if *N*_*e*_ is not larger than a few hundred, even with relatively modest amounts of data. Estimation is more challenging when *N*_*e*_ is >1000 or so, as then the drift signal is small compared to sampling error. Using large samples of individuals and loci can help to some extent, but obtaining robust estimates when effective size is large remains challenging (Marandel et al., 2019; Wang, 2023; Waples, 2016a, 2021; Waples et al., 2022; Waples, Grewe, et al., 2018). Both single‐sample methods were developed for models that make a number of simplifying assumptions, and sensitivities to many of these assumptions have been evaluated; see Wang (2016) and Waples (2024) for details.

The temporal method (Nei & Tajima, 1981; Wang, 2001; Waples, 1989) also was developed for species with discrete generations. However, estimates generally have high uncertainty unless several generations have elapsed between samples (Luikart et al., 2010), in which case estimating the *N*_*e*_/*N* ratio can be problematical unless abundance has been constant. For semelparous Pacific salmon, Waples (1990) developed (and Tajima, 1992 and Waples et al., 2007 refined) a method to estimate *N*_*e*_ per generation based on genetic samples taken one or a few years apart. This method requires information on the distribution of age at maturity and expected fecundity at age for each sex. If samples from at least 3 years are available, it is possible to estimate *N*_*b*_ for individual years using the salmon‐modified temporal method (see King et al., 2023 for a recent example).

Single‐sample estimators are particularly useful for rapid detection of population bottlenecks or fragmentation, provided that a series of temporal samples is available (England et al., 2010; Luikart et al., 2010; Schweizer et al., 2021; Tallmon et al., 2010).

#### 4.2.2 *N*_*e*_ per generation

##### Demographic estimates

The most reliable way to estimate *N*_*e*_ per generation in iteroparous species is to collect enough longitudinal demographic data to calculate variance in LRS for members of a cohort, with the result being used in Equation 13. To the extent that any positive or negative temporal autocorrelations in individual reproductive success occur, their effects will be seen in $\sigma_{k•}^{2}$, and Hill's model reliably predicts the rate of genetic drift even under extreme reproductive scenarios (Waples, 2023b). Of course, collecting the necessary data to make these calculations is easier said than done. For many species, adult lifespan will exceed the duration of a Ph.D thesis, and for some long‐lived ones, it can exceed entire scientific lifetimes. Another, less demanding, option is to use age‐specific vital rates from a life table, including age‐specific estimates of *ϕ*. With these data, using AgeNe, it is possible to extrapolate from annual reproduction to LRS, provided one assumes that within individuals there are no temporal autocorrelations in RS.

A third demographic alternative is to use Nunney and Elam's (1994) “minimal” method, which requires estimates of 9 parameters: the adult sex ratio, and (for both males and females): age at maturity, adult lifespan, generation length, and annual variance in offspring number.

##### Genetic estimates

Estimating *N*_*e*_ when generations overlap is challenging using any of the available genetic methods. The standard temporal method assumes discrete generations; the main challenge in applying it to iteroparous species is in estimating the population's allele frequency at discrete points in time. Logistical challenges in doing this introduce bias that is minimized by sampling across many generations (generally 3–5 at a minimum), at which point the drift signal become relatively large compared to sampling error (Waples & Yokota, 2007). This, however, makes it difficult to estimate *N*_*e*_/*N* for individual generations. Jorde and Ryman (1996) and Jorde (2012) developed a variation of the temporal method that is designed to estimate *N*_*e*_ by sampling from the population at intervals of one to a few years. This method requires estimates of age‐specific survival and fecundity and assumes that the population is stable in size over the time period sampled. It has been evaluated for scenarios in which only some of the adults participate in reproduction each year (e.g., due to skip breeding) but not for scenarios where *ϕ* > 1.

The two most widely used single‐sample genetic estimators (the LD and sibship methods; Palstra & Fraser, 2012) also assume discrete generations. Wang et al. (2010) proposed a variation of the sibship method that is applicable to overlapping generations, provided one knows the sex and age of every individual sampled. However, this method assumes that *ϕ* = 1 for each age and sex, which limits its generality. Age structure affects the amount of LD in a sample and hence LD‐based estimates. In a sample of offspring from a single cohort, the primary drift signal is new LD created by a finite number of parents (hence a signal of *N*_*b*_), but residual LD that has not yet broken down depends on *N*_*e*_ per generation. Thus, the raw estimate of annual *N*_*b*_ is biased to the extent that *N*_*e*_ differs from *N*_*b*_. This bias can be adjusted for based on the ratio *N*_*b*_/*N*_*e*_, which can be estimated using 2 or 3 simple life history traits (Waples et al., 2014). An estimate of *N*_*b*_ can then be converted to *N*_*e*_ using the same estimated ratio *N*_*b*_/*N*_*e*_. Generational *N*_*e*_ can also be estimated directly using LD, but with more uncertainty. Waples et al. (2014) modeled 20 iteroparous species sampled randomly from all adults and found that ${\hat{N}}_{e}$ was always less than true *N*_*e*_, by ~10–50%.

Genetic methods to estimate either annual or generational effective size can produce infinitely large estimates. In the temporal or LD methods, this occurs when the value of the index (temporal *F* or *r* ^2^) is no larger than what would be expected from sampling error alone; in the sibship method, this occurs when no siblings are identified—both results that are expected for populations of infinite size. A similar phenomenon can occur with estimates of population differentiation. Unbiased estimators of *F*_ST_ (e.g., Weir & Cockerham, 1984) can be negative if the magnitude of allele frequency difference is less than would be expected from sampling the same population two or more times. If true *N*_*e*_ is large (>1000, say), infinite point estimates can be common unless a great deal of data (large samples and large numbers of loci) are available. With more modest amounts of data, infinite estimates can also occur when true *N*_*e*_ is moderately large (100s). Many researchers are tempted to ignore infinite *N*_*e*_ estimates, but that is a mistake and can lead to bias because these actually are the largest estimates (see discussion in Waples, 2024). Although infinite *N*_*e*_ is of course impossible in any real population, the lower bound to the confidence intervals for infinite ${\hat{N}}_{e}$ and ${\hat{N}}_{e}/N$ can be informative for conservation and management.

### 4.3 Precision

The most common genetic estimators of *N*_*e*_ or *N*_*b*_ all have robust methods for generating confidence intervals, and these are widely applied. Curiously, although methods to calculate confidence intervals for abundance estimates are also widely available, uncertainty in $\hat{N}$ is not routinely considered in evaluating *N*_*e*_/*N* ratios (Palstra & Fraser, 2012). Ratios are tricky to evaluate statistically because they generally are not normally distributed and can become arbitrarily large as the denominator approaches zero. Furthermore, the variance of a ratio depends not only on means and variances of both the numerator and the denominator, but also on the covariance between them. I agree with Palstra and Fraser (2012) that considerable improvements are needed to develop better ways to characterize uncertainty in *N*_*e*_/*N* and *N*_*b*_/*N*.

## 5 RANGES OF THE *N*_*E*_ /*N* RATIO

### 5.1 Theoretical considerations

Because sex‐ratio skew must be fairly extreme to strongly affect *N*_*e*_ (Section 2.3.3), focus in this section is on variance in offspring number.

#### 5.1.1 Annual reproduction

Family‐correlated mortality, whether due to bad luck or bad genes, increases $\sigma_{k}^{2}$ and reduces *N*_*b*_/*N*. If reproduction is random but subsequently entire families of offspring either survive or not as a unit, then *N*_*b*_/*N* is just the survival rate through the correlated‐mortality stage (Waples, 2002a). The expected value of *N*_*b*_/*N* can also be predicted from the distribution of parental weights (Equation 8), which reflect a combination of successful reproduction by the parents and survival of their offspring until age at enumeration.

Null parents (those that produce no offspring) play an interesting role in effective size: census size ratios. It has become increasingly common in the published literature to find statements to the effect that *N*_*e*_ is the “breeding population” or the number of parents that produce at least one offspring. That this is not universally true is easily seen by considering a Wright–Fisher ideal population, which on average has *N*_*b*_ = *N* but also typically has 13%–14% null parents. Additional factors that can create discrepancies between *N*_*b*_ and the number of successful parents are discussed in Waples (2022a). Nevertheless, in some cases, this rule‐of‐thumb will be approximately correct. In his analysis of large variance in reproductive success, Hedrick (2005) found that if a small number (*N+*) of *N* potential parents are very successful and the rest null, *N*_*b*_/*N* is approximately *N+*/*N*, and this is true whether the successful parents have identical or Poisson variation in offspring number. On the other hand, Hedrick also showed that when a substantial number of null parents were allowed (in the model) to produce on average 2 offspring each, *N+* increased substantially but *N*_*b*_ remained largely unchanged. A similar phenomenon can be seen by comparing the last two columns in Table 1. In Scenario G, only 2 of 10 potential parents produce any offspring (with individual 1 producing twice as many as individual 2), and *N*_*e*_ is only 1.8. In Scenario H, 6 more individuals produce 1 offspring each (so only 2 potential parents are null), but this has little effect on *N*_*e*_, which increases only to 2.

#### 5.1.2 Lifetime reproduction

In a landmark series of papers, Nunney (1991, 1993, 1995, 1996) argued that under most realistic life history scenarios for species with overlapping generations, *N*_*e*_/*N* should converge on ~0.5 as longevity increased, and rather special conditions are required for the ratio to be much below 0.5. These evaluations, however, did not consider all factors that can affect *N*_*e*_ according to Equation 13. Waples (2016b) evaluated life history traits that affect the three key parameters that influence *N*_*e*_/*N* according to Hill's model (*T*, $\sigma_{k•}^{2}$, and *N*), and when considering only longevity obtained results consistent with Nunney's. However, consideration of other factors suggests a wider range of plausible outcomes. Inspection of Equation 13 indicates that Hill's *N*_*e*_ largely reflects a contrast between effects of generation length (*T*) in the numerator and lifetime variance in offspring number ($\sigma_{k•}^{2}$) in the denominator. A number of factors, including adult mortality, have similar effects on both *T* and $\sigma_{k•}^{2}$, resulting in little net effect on *N*_*e*_ or *N*_*e*_/*N*. Two factors, however, are qualitatively different in this respect. First, delayed age at maturity (*α* > 1) increases *T* but by itself does not affect adult lifespan or $\sigma_{k•}^{2}$, so the net effect is to increase *N*_*e*_/*N*, such that the latter can be >1, even with Poisson variation in reproductive success. Second, overdispersed variance in reproductive success among individuals of the same age and sex (*ϕ*_*x*_ > 1) increases $\sigma_{k•}^{2}$ but has no effect on *T*. In fact, the only plausible way to generate “tiny” *N*_*e*_/*N* ratios (<10^−2^ or 10^−3^) is for age‐specific *ϕ* values to be very large (Waples, 2016b).

Finally, in iteroparous species, *N*_*e*_/*N* is influenced by temporal autocorrelations in individual reproductive success, with skip breeding increasing the ratio and persistent individual differences reducing it. For results of simulations that explored effects of positive and negative autocorrelations on $\sigma_{k•}^{2}$ and *N*_*e*_/*N*, see Waples (2023b).

### 5.2 Empirical estimates

Empirical estimates of *N*_*e*_/*N* are plagued by several systematic problems, including (1) inconsistent definitions of *N*; (2) failure to distinguish between *N*_*b*_ and *N*_*e*_; and (3) improperly matching ${\hat{N}}_{e}$ and $\hat{N}$ for different time periods. Nevertheless, results of several large meta‐analyses are informative. Frankham (1995) reported a mean of 0.35 for single‐generation estimates that considered both sex ratio and variance in offspring number, using both demographic and genetic methods. Frankham (1995) also reported a mean “comprehensive” estimate of ~0.1 for multigeneration *N*_*e*_/*N*, which has been widely cited, but this was only obtained by mixing harmonic and arithmetic means (see Table 2). Palstra and Fraser (2012) found median values of both *N*_*e*_/*N* and *N*_*b*_/*N* to be 0.23 for estimates they considered to have properly matched effective and census sizes. They only considered genetic estimates of effective size, but these medians include both single‐generation and multigeneration estimates. Clarke et al. (2024) restricted their analysis to single‐sample genetic estimates using the LD method or the sibship method. They found that median *N*_*e*_/*N* estimates varied by taxonomic group, ranging from 0.09 for invertebrates to 0.48 for birds, with an overall median of 0.24.

Ratios like *N*_*e*_/*N* are typically not normally distributed, which presents challenges for calculating a central tendency when a series of estimates is available. Table 2 illustrates some common approaches with a hypothetical series of eight estimates. The median is often a good choice if enough datapoints are available. A good alternative for ratios is the geometric mean, which is the square root of the product of the arithmetic and harmonic means.

**FIGURE 6:**
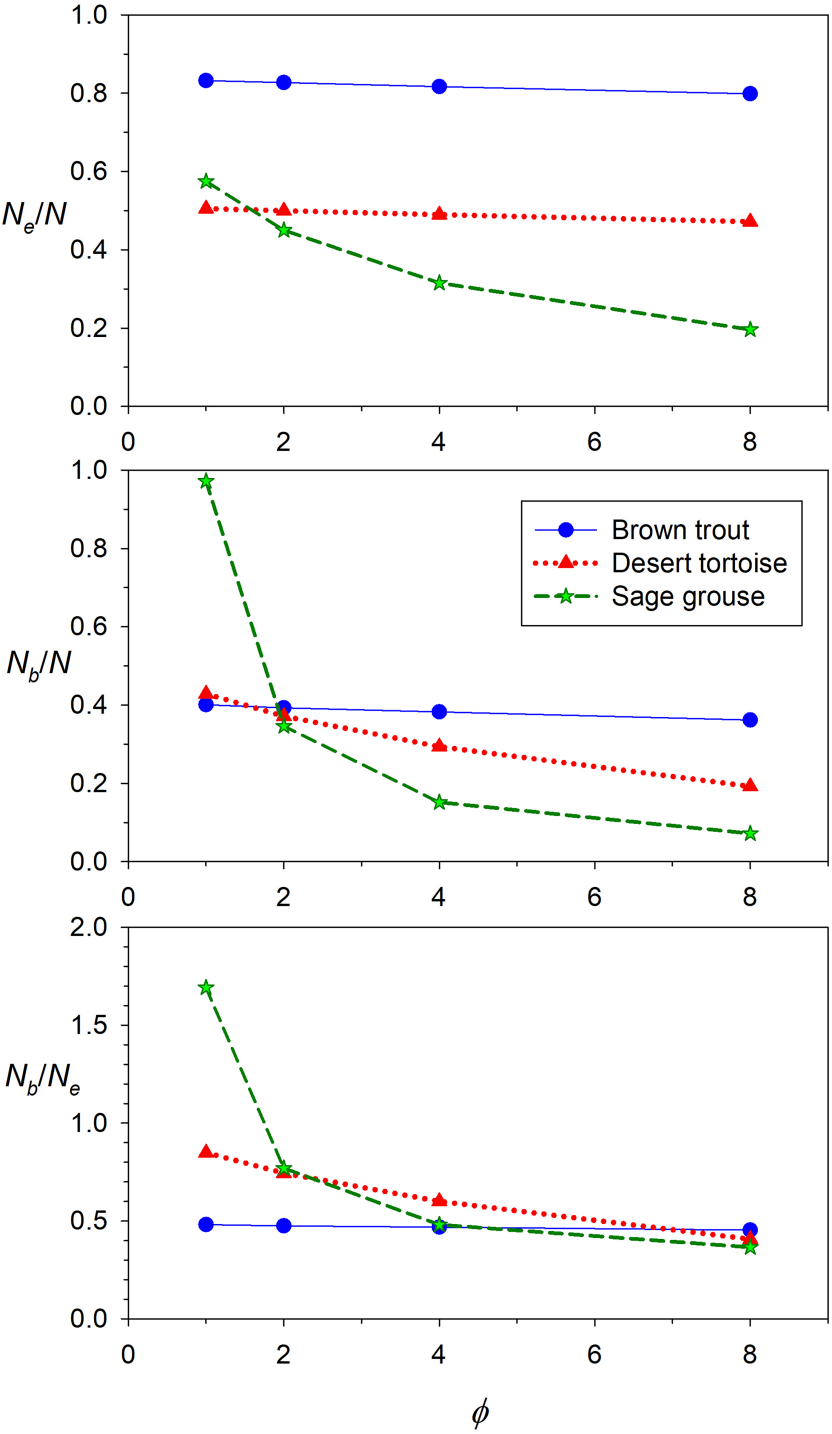
Effects on key effective size ratios of increasing the value of ${\phi_{x}=\sigma }_{k,x}^{2}/b_{x}$ for each age from 1 (indicating Poisson variance in offspring number) to 8 (such that variance is 8 times the mean). Results are based on published vital rates for three species of conservation concern. Top: *Y* axis shows the ratio *N*_*e*_/*N*; Middle: *Y* axis shows the ratio *N*_*b*_/*N*; Bottom: *Y* axis shows the ratio *N*_*b*_/*N*_*e*_. These results were obtained by using the age‐specific vital rates in Table S2 as inputs to the program AgeNe (Waples et al., 2011). For more details, see Waples et al. (2013).

A different approach to *N*_*e*_/*N* ratios was adopted by Waples et al. (2013), who used age‐specific vital rates from published life tables to estimate key effective size ratios in 63 diverse species (vertebrates, invertebrates, and plants). Median ratios across all species were 0.68 for *N*_*e*_/*N*, 0.78 for *N*_*b*_/*N*, and 1.08 for *N*_*b*_/*N*_*e*_. A limitation of this study is that age‐specific variances in offspring number were reported for only a few species, so for consistency, it was assumed that *ϕ* = 1 for each age and sex. High *ϕ* reduces both *N*_*b*_ and *N*_*e*_ but generally affects *N*_*b*_ more strongly (Figure 6), so all of these median estimates are biased upward to an unknown degree. In addition, estimates of *N*_*e*_ based on annual vital rates were based on the assumption that individual survival and reproduction were uncorrelated over time.

Within a wide range of different species, researchers have reported an inverse correlation between estimated *N*_*e*_/*N* and population size (Alvarez et al., 2015; Ardren & Kapuscinski, 2003; Ficetola et al., 2010; Pray et al., 1996; Saarinen et al., 2010; Watts et al., 2013). This suggests that reproductive compensation (reduced variance in reproductive success at low density) is a widespread phenomenon. This in turn argues for caution in assuming that any given species can be characterized by a single *N*_*e*_/*N* or *N*_*b*_/*N* ratio. Good examples of large‐scale studies that have generated extensive series of empirical *N*_*e*_/*N* or *N*_*b*_/*N* estimates include Whiteley et al. (2015), Bernos and Fraser (2016), and Ruzzante et al. (2016).

## 6 SUMMARY AND RECOMMENDATIONS

Here are the most important conclusions from the above review/synthesis regarding the *N*_*e*_/*N* ratio. See Table 3 for a summary of factors that influence this key ratio.

- Recent single‐generation estimates of *N*_*e*_/*N* are the most meaningful and the best predictors of what to expect in the near future.
- For a wide range of applications and experimental designs, inbreeding *N*_*e*_ is simpler to calculate and interpret than variance *N*_*e*_.
- It is crucial to clearly define which individuals are included in the census size (*N*). Defining *N* as the number of adults alive at a given time facilitates comparisons across species. The most important consideration is to be clear and consistent about whether juveniles are included or not.
- The numerator and the denominator of the *N*_*e*_/*N* ratio should be temporally matched to ensure that effective size and census size estimates apply to the same time period(s).
- Most species are age‐structured, so it is important to distinguish between effective size per generation (*N*_*e*_) and the effective number of breeders in one season or year (*N*_*b*_). Both *N*_*e*_ and *N*_*b*_ are important for applied conservation and management.
- For discrete generations or annual reproduction in age‐structured species, the two mechanisms that reduce effective size compared to census size are a skewed sex ratio and overdispersed variance in offspring number ($\sigma_{k}^{2}>\mu_{k}$). Sex ratio must be strongly skewed to have an appreciable effect on *N*_*e*_ or *N*_*b*_, so inflated $\sigma_{k}^{2}$ is generally a more important consideration. Factors that cause annual $\sigma_{k}^{2}$ to exceed the random Poisson expectation ($\mu_{k}$) are changes in fecundity with age and overdispersed variance within ages (*ϕ*_*x*_ > 1), with the latter often being more important. Unfortunately, age‐specific variances are not routinely reported along with age‐specific fecundities in standard life tables, which complicates proper evaluation of this important factor.
- With iteroparity, variation in longevity also contributes to variance in LRS ($\sigma_{k•}^{2}$).
- In iteroparous species, *N*_*e*_ per generation depends on a contrast between generation length (*T*, in the numerator of Hill's Equation 13) and $\sigma_{k•}^{2}$ (in the denominator). A longer lifespan increases both *T* and $\sigma_{k•}^{2}$ so by itself does not strongly affect *N*_*e*_/*N*. Delayed maturity increases *T* but by itself does not affect $\sigma_{k•}^{2}$, so it generally increases *N*_*e*_/*N*. Conversely, overdispersed age‐specific variance (*ϕ*_*x*_ > 1) increases $\sigma_{k•}^{2}$ but has no effect on *T* and hence reduces *N*_*e*_/*N*.
- The lifetime variance $\sigma_{k•}^{2}$ and *N*_*e*_/*N* also can be affected by common biological attributes of natural populations that create temporal autocorrelations in individual reproductive success. Skip breeding creates negative correlations that reduce $\sigma_{k•}^{2}$ and increase *N*_*e*_/*N* because it reduces opportunities for some individuals to consistently dominate offspring production. Positive correlations exacerbate disparities among individuals in LRS and reduce *N*_*e*_/*N*.
- Most local populations are connected to others by some level of migration, so metapopulation *N*_*e*_ is also an important consideration. Over a single generation, migration generally does not strongly affect local metrics of genetic drift (rate of change in allele frequency; magnitude of LD) unless it is high in genetic terms (*m* > 5%–10%). However, the amount of genetic diversity in a local population reflects local *N*_*e*_ only under complete long‐term isolation. With even a small amount of sporadic migration, local genetic diversity is strongly affected by metapopulation *N*_*e*_. This suggests that the *N*_*e*_ ≥ 500 criterion in the 50/500 rule should be evaluated with respect to metapopulation *N*_*e*_, not local *N*_*e*_.
- Estimation of *N*_*e*_ and *N*_*e*_/*N* can be done using demographic or genetic methods, or (ideally) both. Single‐sample genetic methods are the easiest to pair with appropriate estimates of *N* to generate meaningful *N*_*e*_/*N* or *N*_*b*_/*N* ratios. Temporal genetic estimators can provide important information regarding multigenerational *N*_*e*_, but these estimates are more challenging to pair with appropriate estimates of *N*.
- Genetic point estimates of infinity should not be ignored or discarded, as they suggest the population could be large—although this result can also occur with smaller *N*_*e*_ if data are limited. Even with an infinite point estimate, the lower bound to the confidence interval can provide important information for conservation and management.
- Although robust methods to estimate *N* have been in widespread use for many decades, uncertainty in $\hat{N}$ is not consistently considered in interpreting *N*_*e*_/*N* estimates. More work is needed to identify optimal ways to characterize uncertainty in estimates of effective size to census size ratios.
- Caution should be exercised in assuming that effective size: census size ratios are fixed within a species. Empirical data for many species show evidence of reproductive compensation, with reduced $\sigma_{k}^{2}$ and increased *N*_*e*_/*N* or *N*_*b*_/*N* when abundance is relatively low.

**TABLE 3:** Factors that influence effective size and effective size: census size ratios.

Factor	Consequences	Sections	Comments
Definition of *N*	Large effect on both *N*_*b*_/*N* & *N*_*e*_/*N*	2.2	Generally, *N* = number of adults
Uneven sex ratio		Adult sex ratio	Little effect unless skew is strong
Primary	Reduces *N*_*b*_ & *N*_*e*_		Important primarily for species with *TSD*
Adult	Reduces *N*_*b*_ & *N*_*e*_		Arises from unequal survival rates in *M&F*
Variance in offspring number		Variance in offspring number	Potentially very large effect
Family‐correlated survival	Increases $\sigma_{k}^{2}$ & $\sigma_{k•}^{2}$; reduces *N*_*b*_ & *N*_*e*_	3.1, 5.1.1	Common at early life stages
Natural selection	Increases $\sigma_{k}^{2}$ & $\sigma_{k•}^{2}$; reduces *N*_*b*_ & *N*_*e*_	3.2	Magnitude of effect depends on ${\mathrm{CV}}_{W}^{2}$
Changes in *b*_*x*_ with age	Increases $\sigma_{k}^{2}$ & $\sigma_{k•}^{2}$; reduces *N*_*b*_ & *N*_*e*_	3.4, 5.1.2	Strongest effects with increasing fecundity
Long life span	Increases $\sigma_{k•}^{2}$; reduces *N*_*e*_	3.4, 5.1.2	Increase to $\sigma_{k•}^{2}$ largely offset by increase in *T*
Skip breeding	Reduces *N*_*b*_ but increases *N*_*e*_	3.4	Contrasting annual and lifetime consequences
Persistent differences	Increases $\sigma_{k•}^{2}$; reduces *N*_*e*_	3.4, 3.5	Potentially very large effect
Constrained litter size	Reduces $\sigma_{k}^{2}$ & $\sigma_{k•}^{2}$; increases *N*_*b*_ & *N*_*e*_	3.3	Large effects for *LS* = 1; little effect if *LS* ≥4
Mating systems	Various	3.5	Largest effects when some adults excluded from mating
Population structure	Little effect on local *N*_*b*_ or *N*_*e*_ unless migration is high	3.6	Local genetic diversity depends on metapopulation *N*_*e*_, not local *N*_*e*_
Sex reversal	Reduces *N*_*b*_ but little effect on *N*_*e*_	3.7	Across lifetimes, sex reversal compensates for skewed annual sex ratio
Reproductive compensation	Increases *N*_*b*_ /*N* & *N*_*e*_/*N* at low *N*	5.2	Relaxed competition at low density
Captive propagation	Various	3.8	Potentially large effects of various kinds
Increasing adult mortality	Reduces *N*_*e*_ but increases *N*_*e*_/N	3.4	Expected consequence of climate change and harvest
Delayed age at maturity	Increases *N*_*e*_	3.4	Increases *T* without affecting $\sigma_{k•}^{2}$
Semelparity with age structure	*N*_*e*_ is a multiple of *N*_*b*_	3.4	*N*_*e*_ can be >> annual *N*, so use *N*_*T*_ = Σ*N*_*i*_

Abbreviations: *b*_*x*_, age‐specific fecundity; ${\mathrm{CV}}_{W}^{2}$, squared CV of parental weights; *LS*, litter or clutch size; *M*,*F*, male, female; *T*, generation length; *TSD*, temperature‐dependent sex determination.

## CONFLICT OF INTEREST STATEMENT

The author declares no conflict of interest.

## Supporting information


AppendixS1

## Data Availability

This study generated no new data except by simulation. R Code to conduct the simulations is available on Zenodo (https://doi.org/10.5281/zenodo.10966710).
