# Supplementary material for: The Ne /N ratio in applied conservation
Source: Evol Appl. 2024 May 8;17(5):e13695. doi: 10.1111/eva.13695 (PMC11078298; doi:10.1111/eva.13695)
Supplement: Supplementary file 1 — AppendixS1 [file EVA-17-e13695-s001.docx]

**Supporting Information for Waples, R.S. The Ne/N ratio in applied conservation.**

Table S1. Age-specific vital rates used for the scenarios shown in Figure 3. Top: fecundity (*b_x_*). Bottom: ${{}_{x}=\sigma}_{k,x}^{2}/b_{x}$, which in these scenarios was invariant with age at values from 1 to 100.

*b_x_*

Age flat up up2 up3

-----------------------------------------------

1 1 1 1 1

2 1 2 4 8

3 1 3 9 27

4 1 4 16 64

5 1 5 25 125

6 1 6 36 216

7 1 7 49 343

8 1 8 64 512

9 1 9 81 729

10 1 10 100 1000

Age

------------------------------------------------------

1 1 2 5 10 100

2 1 2 5 10 100

3 1 2 5 10 100

4 1 2 5 10 100

5 1 2 5 10 100

6 1 2 5 10 100

7 1 2 5 10 100

8 1 2 5 10 100

9 1 2 5 10 100

10 1 2 5 10 100

Table S2. Age-specific vital rates for 3 species, using different values of . This file can be loaded into AgeNe (Waples et al. 2011) to produce results shown in Figure 6.

Brown trout data from Jorde and Ryman 1996

14 5000 0.5

1 0.480 0 1 0.480 0 1

2 0.480 0 1 0.480 0 1

3 0.480 0 1 0.480 0 1

4 0.480 0 1 0.480 0 1

5 0.480 0 1 0.480 1.9 1

6 0.5 1.9 1 0.5 11.7 1

7 0.5 26.8 1 0.5 17.8 1

8 0.5 43.7 1 0.5 26.7 1

9 0.5 65.5 1 0.5 34.4 1

10 0.5 73.7 1 0.5 40.7 1

11 0.5 72.7 1 0.5 67.6 1

12 0.5 72.7 1 0.5 67.6 1

13 0.5 72.7 1 0.5 67.6 1

14 0 72.7 1 0 67.6 1

grouse

15 200 0.5

1 0.731 0.55 1 0.731 0.55 1

2 0.733 0.817 1 0.733 0.817 1

3 0.733 0.817 1 0.733 0.817 1

4 0.733 0.817 1 0.733 0.817 1

5 0.733 0.817 1 0.733 0.817 1

6 0.733 0.817 1 0.733 0.817 1

7 0.733 0.817 1 0.733 0.817 1

8 0.733 0.817 1 0.733 0.817 1

9 0.733 0.817 1 0.733 0.817 1

10 0.733 0.817 1 0.733 0.817 1

11 0.733 0.817 1 0.733 0.817 1

12 0.733 0.817 1 0.733 0.817 1

13 0.733 0.817 1 0.733 0.817 1

14 0.733 0.817 1 0.733 0.817 1

15 0.733 0.817 1 0.733 0.817 1

desert tortoise

37 1000 0.5

1 0.716 0.00 1 0.716 0.00 1

2 0.716 0.00 1 0.716 0.00 1

3 0.716 0.00 1 0.716 0.00 1

4 0.721 0.00 1 0.721 0.00 1

5 0.730 0.00 1 0.730 0.00 1

6 0.740 0.00 1 0.740 0.00 1

7 0.752 0.02 1 0.752 0.02 1

8 0.766 0.05 1 0.766 0.05 1

9 0.780 0.11 1 0.780 0.11 1

10 0.794 0.19 1 0.794 0.19 1

11 0.808 0.29 1 0.808 0.29 1

12 0.821 0.41 1 0.821 0.41 1

13 0.833 0.54 1 0.833 0.54 1

14 0.843 0.68 1 0.843 0.68 1

15 0.851 0.81 1 0.851 0.81 1

16 0.857 0.93 1 0.857 0.93 1

17 0.862 1.03 1 0.862 1.03 1

18 0.865 1.13 1 0.865 1.13 1

19 0.867 1.21 1 0.867 1.21 1

20 0.869 1.28 1 0.869 1.28 1

21 0.869 1.34 1 0.869 1.34 1

22 0.870 1.39 1 0.870 1.39 1

23 0.870 1.43 1 0.870 1.43 1

24 0.869 1.46 1 0.869 1.46 1

25 0.869 1.49 1 0.869 1.49 1

26 0.869 1.51 1 0.869 1.51 1

27 0.868 1.53 1 0.868 1.53 1

28 0.868 1.55 1 0.868 1.55 1

29 0.868 1.56 1 0.868 1.56 1

30 0.867 1.57 1 0.867 1.57 1

31 0.867 1.59 1 0.867 1.59 1

32 0.867 1.59 1 0.867 1.59 1

33 0.866 1.60 1 0.866 1.60 1

34 0.866 1.61 1 0.866 1.61 1

35 0.866 1.62 1 0.866 1.62 1

36 0.866 1.62 1 0.866 1.62 1

37 0.866 1.63 1 0.866 1.63 1

Brown trout data from Jorde and Ryman 1996

14 5000 0.5

1 0.480 0 2 0.480 0 2

2 0.480 0 2 0.480 0 2

3 0.480 0 2 0.480 0 2

4 0.480 0 2 0.480 0 2

5 0.480 0 2 0.480 1.9 2

6 0.5 1.9 2 0.5 11.7 2

7 0.5 26.8 2 0.5 17.8 2

8 0.5 43.7 2 0.5 26.7 2

9 0.5 65.5 2 0.5 34.4 2

10 0.5 73.7 2 0.5 40.7 2

11 0.5 72.7 2 0.5 67.6 2

12 0.5 72.7 2 0.5 67.6 2

13 0.5 72.7 2 0.5 67.6 2

14 0 72.7 2 0 67.6 2

grouse

15 200 0.5

1 0.731 0.55 2 0.731 0.55 2

2 0.733 0.817 2 0.733 0.817 2

3 0.733 0.817 2 0.733 0.817 2

4 0.733 0.817 2 0.733 0.817 2

5 0.733 0.817 2 0.733 0.817 2

6 0.733 0.817 2 0.733 0.817 2

7 0.733 0.817 2 0.733 0.817 2

8 0.733 0.817 2 0.733 0.817 2

9 0.733 0.817 2 0.733 0.817 2

10 0.733 0.817 2 0.733 0.817 2

11 0.733 0.817 2 0.733 0.817 2

12 0.733 0.817 2 0.733 0.817 2

13 0.733 0.817 2 0.733 0.817 2

14 0.733 0.817 2 0.733 0.817 2

15 0.733 0.817 2 0.733 0.817 2

desert tortoise

37 1000 0.5

1 0.716 0.00 2 0.716 0.00 2

2 0.716 0.00 2 0.716 0.00 2

3 0.716 0.00 2 0.716 0.00 2

4 0.721 0.00 2 0.721 0.00 2

5 0.730 0.00 2 0.730 0.00 2

6 0.740 0.00 2 0.740 0.00 2

7 0.752 0.02 2 0.752 0.02 2

8 0.766 0.05 2 0.766 0.05 2

9 0.780 0.11 2 0.780 0.11 2

10 0.794 0.19 2 0.794 0.19 2

11 0.808 0.29 2 0.808 0.29 2

12 0.821 0.41 2 0.821 0.41 2

13 0.833 0.54 2 0.833 0.54 2

14 0.843 0.68 2 0.843 0.68 2

15 0.851 0.81 2 0.851 0.81 2

16 0.857 0.93 2 0.857 0.93 2

17 0.862 1.03 2 0.862 1.03 2

18 0.865 1.13 2 0.865 1.13 2

19 0.867 1.21 2 0.867 1.21 2

20 0.869 1.28 2 0.869 1.28 2

21 0.869 1.34 2 0.869 1.34 2

22 0.870 1.39 2 0.870 1.39 2

23 0.870 1.43 2 0.870 1.43 2

24 0.869 1.46 2 0.869 1.46 2

25 0.869 1.49 2 0.869 1.49 2

26 0.869 1.51 2 0.869 1.51 2

27 0.868 1.53 2 0.868 1.53 2

28 0.868 1.55 2 0.868 1.55 2

29 0.868 1.56 2 0.868 1.56 2

30 0.867 1.57 2 0.867 1.57 2

31 0.867 1.59 2 0.867 1.59 2

32 0.867 1.59 2 0.867 1.59 2

33 0.866 1.60 2 0.866 1.60 2

34 0.866 1.61 2 0.866 1.61 2

35 0.866 1.62 2 0.866 1.62 2

36 0.866 1.62 2 0.866 1.62 2

37 0.866 1.63 2 0.866 1.63 2

Brown trout data from Jorde and Ryman 1996

14 5000 0.5

1 0.480 0 4 0.480 0 4

2 0.480 0 4 0.480 0 4

3 0.480 0 4 0.480 0 4

4 0.480 0 4 0.480 0 4

5 0.480 0 4 0.480 1.9 4

6 0.5 1.9 4 0.5 11.7 4

7 0.5 26.8 4 0.5 17.8 4

8 0.5 43.7 4 0.5 26.7 4

9 0.5 65.5 4 0.5 34.4 4

10 0.5 73.7 4 0.5 40.7 4

11 0.5 72.7 4 0.5 67.6 4

12 0.5 72.7 4 0.5 67.6 4

13 0.5 72.7 4 0.5 67.6 4

14 0 72.7 4 0 67.6 4

grouse

15 200 0.5

1 0.731 0.55 4 0.731 0.55 4

2 0.733 0.817 4 0.733 0.817 4

3 0.733 0.817 4 0.733 0.817 4

4 0.733 0.817 4 0.733 0.817 4

5 0.733 0.817 4 0.733 0.817 4

6 0.733 0.817 4 0.733 0.817 4

7 0.733 0.817 4 0.733 0.817 4

8 0.733 0.817 4 0.733 0.817 4

9 0.733 0.817 4 0.733 0.817 4

10 0.733 0.817 4 0.733 0.817 4

11 0.733 0.817 4 0.733 0.817 4

12 0.733 0.817 4 0.733 0.817 4

13 0.733 0.817 4 0.733 0.817 4

14 0.733 0.817 4 0.733 0.817 4

15 0.733 0.817 4 0.733 0.817 4

desert tortoise

37 1000 0.5

1 0.716 0.00 4 0.716 0.00 4

2 0.716 0.00 4 0.716 0.00 4

3 0.716 0.00 4 0.716 0.00 4

4 0.721 0.00 4 0.721 0.00 4

5 0.730 0.00 4 0.730 0.00 4

6 0.740 0.00 4 0.740 0.00 4

7 0.752 0.02 4 0.752 0.02 4

8 0.766 0.05 4 0.766 0.05 4

9 0.780 0.11 4 0.780 0.11 4

10 0.794 0.19 4 0.794 0.19 4

11 0.808 0.29 4 0.808 0.29 4

12 0.821 0.41 4 0.821 0.41 4

13 0.833 0.54 4 0.833 0.54 4

14 0.843 0.68 4 0.843 0.68 4

15 0.851 0.81 4 0.851 0.81 4

16 0.857 0.93 4 0.857 0.93 4

17 0.862 1.03 4 0.862 1.03 4

18 0.865 1.13 4 0.865 1.13 4

19 0.867 1.21 4 0.867 1.21 4

20 0.869 1.28 4 0.869 1.28 4

21 0.869 1.34 4 0.869 1.34 4

22 0.870 1.39 4 0.870 1.39 4

23 0.870 1.43 4 0.870 1.43 4

24 0.869 1.46 4 0.869 1.46 4

25 0.869 1.49 4 0.869 1.49 4

26 0.869 1.51 4 0.869 1.51 4

27 0.868 1.53 4 0.868 1.53 4

28 0.868 1.55 4 0.868 1.55 4

29 0.868 1.56 4 0.868 1.56 4

30 0.867 1.57 4 0.867 1.57 4

31 0.867 1.59 4 0.867 1.59 4

32 0.867 1.59 4 0.867 1.59 4

33 0.866 1.60 4 0.866 1.60 4

34 0.866 1.61 4 0.866 1.61 4

35 0.866 1.62 4 0.866 1.62 4

36 0.866 1.62 4 0.866 1.62 4

37 0.866 1.63 4 0.866 1.63 4

Brown trout data from Jorde and Ryman 1996

14 5000 0.5

1 0.480 0 8 0.480 0 8

2 0.480 0 8 0.480 0 8

3 0.480 0 8 0.480 0 8

4 0.480 0 8 0.480 0 8

5 0.480 0 8 0.480 1.9 8

6 0.5 1.9 8 0.5 11.7 8

7 0.5 26.8 8 0.5 17.8 8

8 0.5 43.7 8 0.5 26.7 8

9 0.5 65.5 8 0.5 34.4 8

10 0.5 73.7 8 0.5 40.7 8

11 0.5 72.7 8 0.5 67.6 8

12 0.5 72.7 8 0.5 67.6 8

13 0.5 72.7 8 0.5 67.6 8

14 0 72.7 8 0 67.6 8

grouse

15 200 0.5

1 0.731 0.55 8 0.731 0.55 8

2 0.733 0.817 8 0.733 0.817 8

3 0.733 0.817 8 0.733 0.817 8

4 0.733 0.817 8 0.733 0.817 8

5 0.733 0.817 8 0.733 0.817 8

6 0.733 0.817 8 0.733 0.817 8

7 0.733 0.817 8 0.733 0.817 8

8 0.733 0.817 8 0.733 0.817 8

9 0.733 0.817 8 0.733 0.817 8

10 0.733 0.817 8 0.733 0.817 8

11 0.733 0.817 8 0.733 0.817 8

12 0.733 0.817 8 0.733 0.817 8

13 0.733 0.817 8 0.733 0.817 8

14 0.733 0.817 8 0.733 0.817 8

15 0.733 0.817 8 0.733 0.817 8

desert tortoise

37 1000 0.5

1 0.716 0.00 8 0.716 0.00 8

2 0.716 0.00 8 0.716 0.00 8

3 0.716 0.00 8 0.716 0.00 8

4 0.721 0.00 8 0.721 0.00 8

5 0.730 0.00 8 0.730 0.00 8

6 0.740 0.00 8 0.740 0.00 8

7 0.752 0.02 8 0.752 0.02 8

8 0.766 0.05 8 0.766 0.05 8

9 0.780 0.11 8 0.780 0.11 8

10 0.794 0.19 8 0.794 0.19 8

11 0.808 0.29 8 0.808 0.29 8

12 0.821 0.41 8 0.821 0.41 8

13 0.833 0.54 8 0.833 0.54 8

14 0.843 0.68 8 0.843 0.68 8

15 0.851 0.81 8 0.851 0.81 8

16 0.857 0.93 8 0.857 0.93 8

17 0.862 1.03 8 0.862 1.03 8

18 0.865 1.13 8 0.865 1.13 8

19 0.867 1.21 8 0.867 1.21 8

20 0.869 1.28 8 0.869 1.28 8

21 0.869 1.34 8 0.869 1.34 8

22 0.870 1.39 8 0.870 1.39 8

23 0.870 1.43 8 0.870 1.43 8

24 0.869 1.46 8 0.869 1.46 8

25 0.869 1.49 8 0.869 1.49 8

26 0.869 1.51 8 0.869 1.51 8

27 0.868 1.53 8 0.868 1.53 8

28 0.868 1.55 8 0.868 1.55 8

29 0.868 1.56 8 0.868 1.56 8

30 0.867 1.57 8 0.867 1.57 8

31 0.867 1.59 8 0.867 1.59 8

32 0.867 1.59 8 0.867 1.59 8

33 0.866 1.60 8 0.866 1.60 8

34 0.866 1.61 8 0.866 1.61 8

35 0.866 1.62 8 0.866 1.62 8

36 0.866 1.62 8 0.866 1.62 8

37 0.866 1.63 8 0.866 1.63 8
